# Relationship between sodium channel function and clinical phenotype in SCN5A variants associated with Brugada syndrome

**DOI:** 10.1002/humu.24128

**Published:** 2020-11-11

**Authors:** Charles M. Pearman, Nathan C. Denham, Robert W. Mills, Wern Y. Ding, Simon S. Modi, Mark C. S. Hall, Derick M. Todd, Saagar Mahida

**Affiliations:** ^1^ Department of Cardiac Electrophysiology and Inherited Cardiac Conditions Liverpool Heart and Chest Hospital Liverpool UK; ^2^ Unit of Cardiac Physiology, Division of Cardiovascular Sciences, Manchester Academic Health Science Centre, Core Technology Facility University of Manchester Manchester UK; ^3^ Cardiovascular Research Center Massachusetts General Hospital Charlestown Massachusetts USA; ^4^ Liverpool Centre for Cardiovascular Science, Faculty of Life Sciences University of Liverpool Liverpool UK

**Keywords:** Brugada, patch clamp, risk stratification, sodium current

## Abstract

The identification of a pathogenic SCN5A variant confers an increased risk of conduction defects and ventricular arrhythmias (VA) in Brugada syndrome (BrS). However, specific aspects of sodium channel function that influence clinical phenotype have not been defined. A systematic literature search identified *SCN5A* variants associated with BrS. Sodium current (*I*_Na_) functional parameters (peak current, decay, steady‐state activation and inactivation, and recovery from inactivation) and clinical features (conduction abnormalities [CA], spontaneous VA or family history of sudden cardiac death [SCD], and spontaneous BrS electrocardiogram [ECG]) were extracted. A total of 561 *SCN5A* variants associated with BrS were identified, for which data on channel function and clinical phenotype were available in 142. In the primary analysis, no relationship was found between any aspect of channel function and CA, VA/SCD, or spontaneous BrS ECG pattern. Sensitivity analyses including only variants graded pathogenic or likely pathogenic suggested that reduction in peak current and positive shift in steady‐state activation were weakly associated with CA and VA/SCD, although sensitivity and specificity remained low. The relationship between in vitro assessment of channel function and BrS clinical phenotype is weak. The assessment of channel function does not enhance risk stratification. Caution is needed when extrapolating functional testing to the likelihood of variant pathogenicity.

## Graphical abstract

A systematic review and meta‐analysis was performed of published SCN5A variants associated with Brugada syndrome to assess the relationship between in vitro effect on sodium channel function and clinical phenotype. The association was weak between a variant's in vitro effect and the incidence of spontaneous ventricular arrhythmias, conduction disease, or a spontaneous Type 1 ECG pattern.

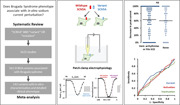

## 1 INTRODUCTION

The manifestations of Brugada syndrome (BrS) range from recurrent life‐threatening ventricular arrhythmias (VA) to merely an asymptomatic abnormality on an electrocardiogram (ECG) seen during provocative testing (Priori et al., 2002). Currently, identification of those at high risk of arrhythmias relies heavily on prior VA, either documented or suggested by symptoms (Priori et al., 2015). Conduction abnormalities (CA) and a spontaneous type 1 BrS ECG have been associated with a greater arrhythmic risk (Sieira et al., 2017). A family history of early sudden cardiac death (SCD) in first degree relatives may also confer a worse prognosis (Sieira et al., 2017), raising the question of whether specific genetic variants may influence risk.

While variants in more than 40 genes have been reported in association with BrS (Campuzano et al., 2019), the most robust evidence for pathogenicity is associated with *SCN5A*, the gene encoding NaV1.5, the α‐subunit of the cardiac sodium channel (Hosseini et al., 2018). In vitro functional assessment has demonstrated that the majority of BrS‐associated *SCN5A* variants lead to attenuation of the sodium current (*I*_Na_), although the extent of loss‐of‐function varies widely (Denham et al., 2018; Glazer et al., 2020). A meta‐analysis exploring the potential role of genetic testing to enhance risk stratification suggests that individuals in whom an *SCN5A* variant can be identified are more likely to have conduction defects, VA, and a spontaneous type 1 ECG (Chen et al., 2019). Recent evidence has emerged suggesting that *SCN5A* variants that cause greater perturbation of *I*_Na_ in vitro are associated with higher BrS penetrance (Kroncke et al., 2018). Accordingly, in vitro assessment of channel function has been included in scoring systems to assess pathogenicity of genetic variants (Denham et al., 2018; Richards et al., 2015). However, it remains uncertain as to whether mild‐to‐moderate perturbation of channel function is sufficient to denote pathogenicity of a variant. Furthermore, there is growing evidence to suggest that BrS is a polygenic trait (Cerrone et al., 2019). The relationship between channel function and aspects of the BrS phenotype such as arrhythmic risk has not been systematically evaluated.

In this study, we performed a systematic analysis of all functionally characterized *SCN5A* variants implicated in BrS to investigate the relationship between functional parameters and clinical phenotype. Specifically, we investigated the association with (1) VA and SCD, (2) manifest CA, and (3) a spontaneous BrS ECG pattern.

## 2 METHODS

### 2.1 Search strategy

PubMed was searched on August 1, 2020 using the search terms “SCN5A” and (“mutation” or “variant”). Previously published reviews, compendia, and online resources (e.g., Kapplinger et al., 2015, 2010; Kroncke et al., 2018) were used to identify additional variants, clinical and functional descriptions. Variants were included if (1) they had been reported in association with a spontaneous and/or drug‐induced type 1 BrS pattern, (2) a detailed clinical phenotype for at least one gene‐positive family member was present, and (3) in vitro functional testing using patch‐clamp electrophysiology had been performed. Variants associated with other phenotypes including long QT syndrome, progressive cardiac conduction disease, and sudden unexplained death syndrome were included only if at least one gene‐positive family member manifested a type 1 BrS pattern.

### 2.2 In vitro functional testing

Continuous data were extracted for difference in peak *I*_Na_ during homozygous and heterozygous expression for variant channels relative to wild‐type, and the shift in half maximal voltages of steady‐state activation and inactivation. Current decay and recovery from inactivation were extracted as categorical data as these variables were not reported in a standardized manner. Where more than one study assessed a variant, precedence was given to those results obtained from mammalian cell types over nonmammalian cell types, those obtained in the presence of beta‐subunit coexpression, and those recorded at physiological temperatures. Where similar methodologies were used in multiple studies of a single variant, the mean values were taken.

### 2.3 Clinical phenotype

Aspects of clinical phenotype were extracted for each variant in the domains of VA/SCD, CA, and spontaneous type 1 BrS ECG pattern. These were not mutually exclusive, allowing variants to manifest in any, all, or none of these domains. The criteria for VA/SCD were at least one genotype‐positive member of the pedigree experiencing spontaneous VA outside the context of electrophysiological studies or provocative testing, and/or one or more incidences of SCD (whether or not genetic testing had been performed in the affected individuals). Variants met the criteria for CA if at least one gene‐positive member of the pedigree had sinus or atrioventricular (AV) node dysfunction. The criteria for spontaneous BrS ECG were at least one spontaneous type 1 BrS ECG pattern in the pedigree (vs. solely drug‐induced BrS ECG patterns). Variants for which clinical details were only available for family members with compound heterozygosity were excluded from analysis. Total pedigree size and number of gene‐positive individuals were summed from all available reports.

### 2.4 Statistical analysis

Statistical tests were performed in Microsoft Excel 2013 and SigmaPlot v7.0 (Systat software). Areas under the curve (AUC) were calculated using a trapezoidal method. The discriminatory power of receiver operating characteristic (ROC) curves was classified based on the AUC, with AUC less than 0.6 interpreted as showing no relationship. Youden's J index was used to calculate the optimal cutoff. Data were tested for normality using the Shapiro–Wilk test. Two‐tailed Student's *t* test was used for comparisons between normally distributed continuous variables, while the Mann–Whitney rank sum test was used for non‐normally distributed data. *χ* ^2^ or Fisher's exact tests were used as appropriate for comparisons between categorical variables. *p* less than .05 was taken as statistical significance. Data are presented as mean ± standard error of the mean.

## 3 RESULTS

The initial search yielded 1625 studies. A total of 561 BrS‐associated *SCN5A* variants were identified, for which in vitro functional data were available for 194. A total of 142 variants had sufficient clinical and functional data to be included in the analysis (missense *n* = 116, premature truncation *n* = 12, frameshift *n* = 6, insertion *n* = 4, and deletion *n* = 4). Variants with functional data but insufficient clinical data were not included in the analysis. Variants were classified by the American College of Medical Genetics and Genomics (ACMG) criteria as pathogenic (*n* = 22), likely pathogenic (*n* = 40), variant of uncertain significance (*n* = 75), likely benign (*n* = 3), or benign (*n* = 2), as previously described (Denham et al., 2018). Variants were reported in 526 gene‐positive individuals in pedigrees totaling 1911 individuals (range, 1–164 per pedigree). A complete list of *SCN5A* variants associated BrS can be found in Supporting Information.

Patch clamp electrophysiology was used for all in vitro assessment of channel function. Mammalian cells lines were used for 139 of 142 (98%) variants (human embryonic kidney [HEK293/TSA201] *n* = 131, human‐induced pluripotent stem cell *n* = 4, human cardiomyocyte [iCell] *n* = 2, and Chinese hamster ovary *n* = 2). Beta subunits were coexpressed in 72 of 142 (51%) variants. Heterozygous expression was assessed for 37 of 142 (26%) variants. Currents were recorded at physiological temperatures in 15 of 142 (11%) variants. Variables assessed included peak current (homozygous channel expression *n* = 140 variants; heterozygous expression *n* = 35), steady‐state activation (*n* = 101), steady‐state inactivation (*n* = 101), recovery from inactivation (*n* = 70), and current decay (*n* = 66).

Twenty‐three variants were reported in two or more studies using a similar expression system. Reproducibility within a single expression system was moderate to good. The results obtained were fully concordant in 13 of 23 of these variants. In 7 of 23 variants, minor differences were seen between studies (<50% absolute difference in current decrease [*n* =5], <5 mV difference in reported gating shift [*n* = 1]). In 3 of 23 variants substantial differences between reports were found (≥50% absolute difference in current decrease [*n* = 1], ≥5 mv difference is reported gating shift [n = 2]).

Twenty‐seven variants were assessed using two or more expression systems. Reproducibility between expression systems was poor to moderate. Results were fully concordant between expression systems in 10 of 27 variants. In 4 of 24 variants, minor differences were seen between expression systems (<50% absolute difference in current decrease [*n* = 4]). In 11 of 24 variants, substantial differences were seen (≥50% absolute difference in current decrease [*n* = 5], ≥5 mv difference is reported gating shift [*n* = 7]).

### 3.1 Ventricular arrhythmias

**Figure 1:**
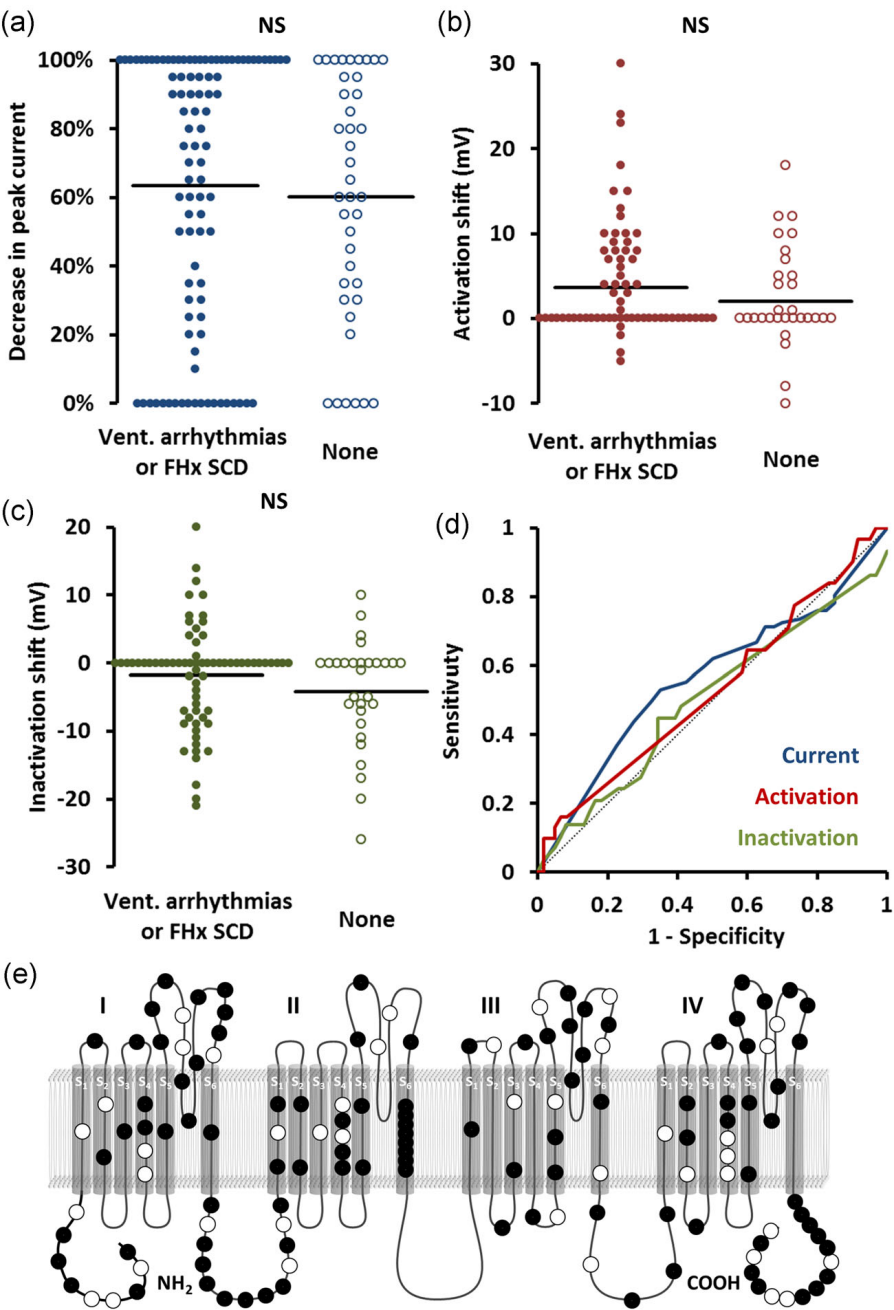
Association with spontaneous ventricular arrhythmias (VA) or family history of sudden cardiac death (SCD). (a–c) Beeswarm plots. Each point represents an*SCN5A*variant plotted against peak current (a), shift in steady‐state activation (b), and shift in steady‐state inactivation (c), stratified by association with VA/SCD. Horizontal bars represent group means. (d) Receiver operating characteristic curves of the same data. Dotted line represents no discrimination. (e) Location of variants on channel. Filled markers represent variants associated with VA/SCD.*NS*, not significant

We first assessed whether the degree of current perturbation was associated with the risk of VA. A total of 101 of 142 (71%) variants were associated with spontaneous VA/SCD in ≥1 family member. Decrease in *I*_Na_ was similar between groups during homozygous expression (VA/SCD+ve 63.4 ± 4.0%; VA/SCD−ve 60.1 ± 5.6%; *n* = 140; *p* = .65, Figure 1a) and heterozygous expression, which takes account of dominant negative effects (VA/SCD+ve 46.3 ± 4.3%; VA/SCD−ve 47.1 ± 3.1%; *n* = 35; *p* = .92). Shifts in steady‐state activation were similar between groups (VA/SCD+ve +3.7 ± 0.3 mV; VA/SCD−ve +2.0 ± 1.1 mV; *n* = 101; *p* = .29, Figure 1b) as were shifts in steady‐state inactivation (VA/SCD+ve −1.7 ± 0.9 mV; VA/SCD−ve −4.2 ± 1.5 mV; *n* = 101; *p* = .13, Figure 1c). Proportions of variants with delayed recovery from inactivation were also comparable between the two groups (VA/SCD+ve 17/48 [35%]; VA/SCD−ve 7/22 [32%]; *n* = 70; *p* = .77). Variants associated with VA were more likely to manifest accelerated current decay, although significance was borderline (VA/SCD+ve 14/49 [29%]; VA/SCD−ve 1/17 [6%]; *n* = 66; *p* = .05).

ROC curve analysis (Figure 1d) showed no significant relationship between VA/SCD and peak current during homozygous (AUC, 0.55) or heterozygous expression (AUC, 0.52), steady‐state activation (AUC, 0.54) or inactivation (AUC, 0.52).

Variants associated with VA/SCD did not localize to a specific region of the channel. Variant distribution is shown in Figure 1e.

### 3.2 Conduction abnormalities

**Figure 2:**
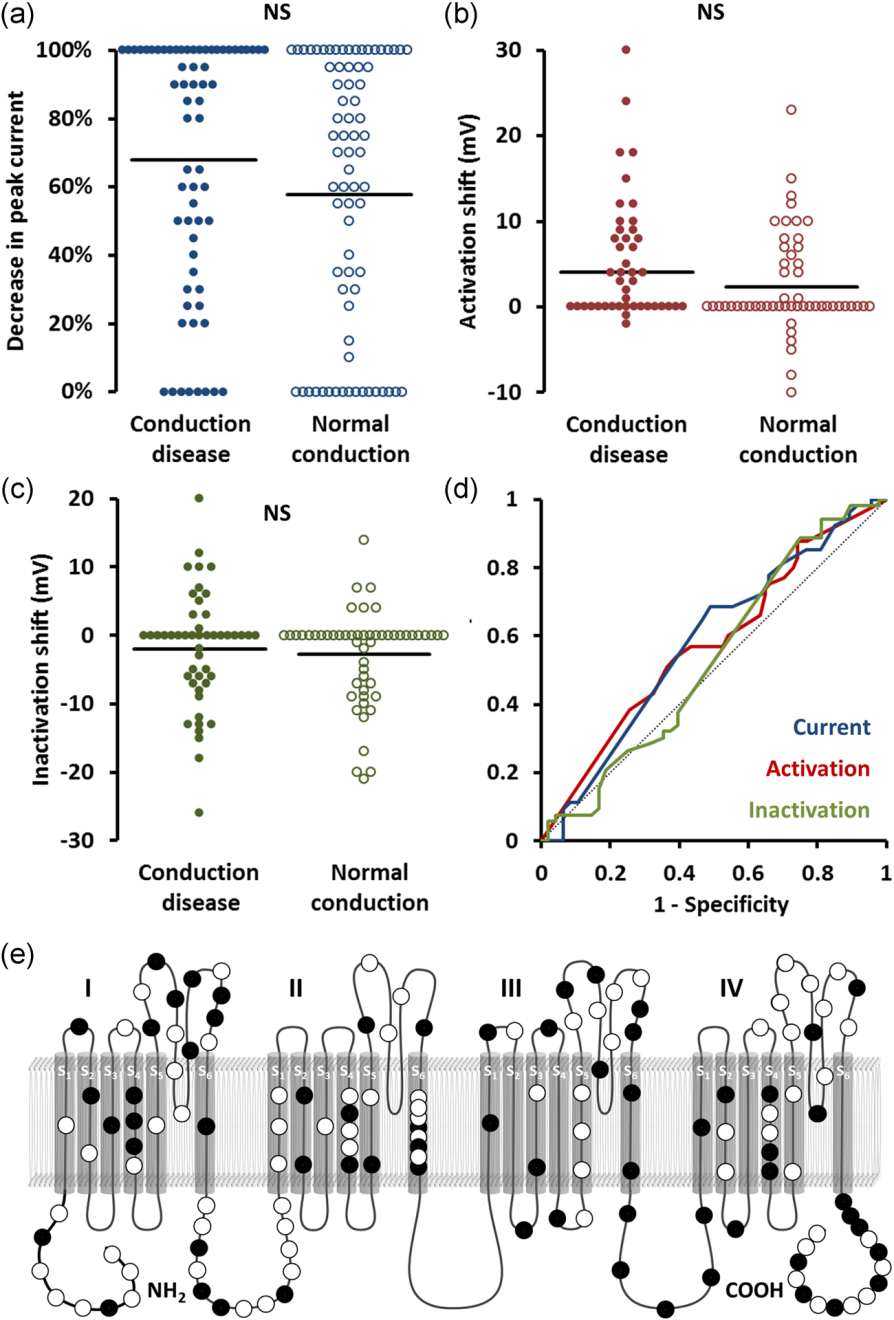
Association with conduction system disease. (a–c) Beeswarm plots. Each point represents an*SCN5A*variant plotted against peak current (a), shift in steady‐state activation (b), and shift in steady‐state inactivation (c), stratified by association with spontaneous ventricular arrhythmias or a family history of sudden cardiac death. Horizontal bars represent group means. (d) Receiver operating characteristic curves of the same data. Dotted line represents no discrimination. (e) Location of variants on channel. Filled markers represent variants associated with conduction disease. *NS*, not significant. *p < .05

We next tested the hypothesis that variants causing a greater perturbation of *I*_Na_ would be more likely to associate with CA. A total of 65 of 142 (46%) variants were associated with one or more CA (sinus node dysfunction, *n* = 32; first‐degree AV block, *n* = 40; high‐grade AV block, *n* = 9; interventricular conduction delay, *n* = 31). Variants associated with CA (CA+ve) had a similar degree of *I*_Na_ reduction compared to those not associated with CA (CA−ve) during heterozygous (CA+ve 68.0 ± 4.5%; CA−ve 57.7 ± 4.6%; *p* = .12, Figure 2a) and homozygous expression (CA+ve 45.8 ± 4.8%; CA−ve 47.1 ± 5.2%; *p* = .86).

Shifts in steady‐state activation (CA+ve +4.1 ± 1.2 mV; CA−ve +2.4 ± 0.8 mV; *p* = .22, Figure 2b) and inactivation (CA+ve −2.0 ± 1.2 mV; CA−ve −2.8 ± 0.9 mV; *p* = .58, Figure 2c) were comparable between the two groups. Similar proportions of variants in each group had evidence of accelerated current decay (CA+ve 7/28 [254%]; CA−ve 8/38 [21%]; *p* = .71) and delayed recovery from inactivation (CA+ve 13/31 [42%]; CA−ve 11/39 [28%]; *p* = .23). Similar results were seen when subtypes of conduction abnormality were assessed separately—no significant associations were seen between any aspect of channel function and sinus node dysfunction, any AV block, or high‐grade AV block.

ROC curves (Figure 1d) showed no significant relationship between CA and peak current during homozygous (AUC, 0.58) or heterozygous expression (AUC, 0.52), steady‐state activation (AUC, 0.58), or inactivation (AUC, 0.53).

Variants associated with CA did not localize to a specific region of the channel. Variant distribution is shown in Figure 2e.

### 3.3 Spontaneous type 1 ECG pattern

**Figure 3:**
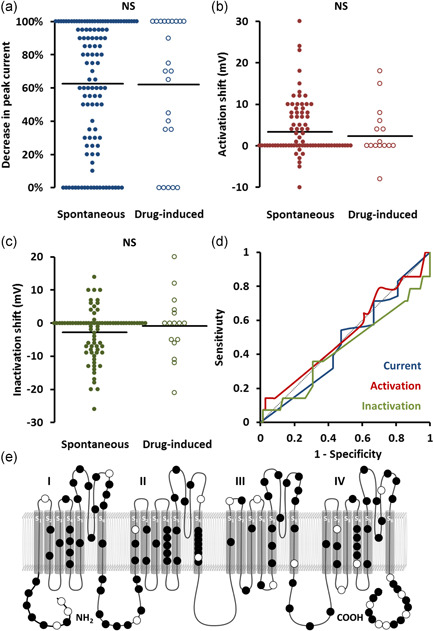
Association with spontaneous type 1 Brugada ECG pattern. (a–c) Beeswarm plots. Each point represents an*SCN5A*variant plotted against peak current (a), shift in steady‐state activation (b), and shift in steady‐state inactivation (c), stratified by association with spontaneous ventricular arrhythmias or a family history of sudden cardiac death. Horizontal bars represent group means. (d) Receiver operating characteristic curves of the same data. Dotted line represents no discrimination. (e) Location of variants on channel. Filled markers represent variants associated with spontaneous type 1 Brugada ECG pattern. ECG, electrocardiogram; *NS*, not significant

As the presence of a spontaneous BrS ECG is felt to represent a more pronounced phenotype, we next explored the association between spontaneous ECG and channel function. A spontaneous type 1 BrS ECG (in ≥1 variant carrier) was described in association with 105 of 142 (74%) variants while drug‐induced type 1 ECG patterns were described for 23 of 142 (16%) variants. The ECG pattern was not clearly described for the remaining 14 variants. Neither decrease in peak *I*_Na_ during homozygous (spontaneous 62.6 ± 3.6%; drug‐induced 62.0 ± 8.4%; *p* = .94, Figure 3a) or heterozygous expression (spontaneous 45.3 ± 4.0%; drug‐induced 53.0 ± 3.0%; *p* = .44), steady‐state activation (spontaneous +3.3 ± 079 mV; drug‐induced +2.3 ± 1.8 mV; *p* = .60, Figure 3b) nor inactivation (spontaneous −2.7 ± 0.8 mV; drug‐induced −0.9 ± 2.4 mV; *p* = .37, Figure 3c) differed between groups. Similar proportions within each group had evidence of accelerated current decay (spontaneous 13/57 [22%]; drug‐induced 1/6 [22%]; *p* = .73) and delayed recovery from inactivation (spontaneous 21/58 [36%]; drug‐induced 3/12 [25%]; *p* = .46).

ROC curve analysis (Figure 3d) showed no significant relationship between spontaneous BrS ECG and peak current during homozygous (AUC, 0.51) or heterozygous expression (AUC, 0.52), steady‐state activation (AUC, 0.53), or inactivation (AUC, 0.43).

Variants associated with a spontaneous BrS ECG did not localize to a specific region of the channel. Variant distribution is shown in Figure 3e.

### 3.4 Sensitivity analyses

As some variants associated with BrS may not be truly causative, we performed a sensitivity analysis including only variants graded as pathogenic or likely pathogenic (according to ACMG scoring). In this subset of 62 variants, ROC analysis showed a modest association between decrease in peak current and CA (AUC, 0.62, optimal cutoff 85% current reduction with sensitivity 78%, and specificity 47%) and VA/SCD (AUC, 0.63, optimal cutoff 85% current reduction with sensitivity 74%, and specificity 54%) with differences in means that approached statistical significance (CA+ve 90.0 ± 3.1%; CA−ve 78.8 ± 4.9%; *p* = .056) and VA/SCD (VA/SCD+ve 87.9 ± 3.0%; VA/SCD−ve 75.0 ± 6.8%; *p* = .052). No significant relationships were seen with any other aspects of channel function or clinical phenotype.

Recognizing that some variants manifested abnormalities in peak current but not gating or vice versa, we attempted to separate out these forms of channel dysfunction. To look at reduction in peak current alone, a sensitivity analysis was performed excluding variants that manifested shifts in steady‐state activation of ≥+5 mV or steady‐state inactivation of ≤−5 mV. In this subanalysis of 88 variants, CA were associated with a greater reduction in peak *I*_Na_ (CA+ve 83.5 ± 4.9%; CA−ve 65.3 ± 6.0%; *p* = .02). ROC analysis showed a modest association between decrease in peak *I*_Na_ and CA (AUC, 0.63, optimal cutoff 95% current reduction, sensitivity 62%, and specificity 61%). No significant association was seen between VA/SCD or spontaneous BrS ECG and any aspect of channel function. To explore changes in gating kinetics alone, a sensitivity analysis was performed excluding variants with greater than 50% decrease in peak *I*_Na_. In this analysis of 52 variants, a positive shift in steady‐state activation was modestly associated with CA (AUC, 0.68, optimal cutoff +1 mV, sensitivity 76%, and specificity 67%) and VA/SCD (AUC, 0.60, optimal cutoff +8 mV, sensitivity 100%, and specificity 21%). Differences in means approached statistical significance for CA (CA+ve 4.0 ± 1.9 mV; CA−ve 0.8 ± 0.8 mV; *p* = .08) but not VA/SCD (VA/SCD+ve 3.0 ± 1.2 mV; VA/SCD−ve 0.3.0 ± 1.3%; *p* = .17).

To remove the confounding effect of variants manifesting an overlapping phenotype of BrS with Long QT syndrome, we next performed a sensitivity analysis that excluded these variants. Excluding 19 variants associated with an overlapping phenotype did not affect the relationships between channel function and CA or spontaneous ECG but, somewhat implausibly, suggested that VA/SCD was associated with a lesser degree of shift in steady‐state inactivation (VA/SCD+ve −0.3 ± 0.9 mV; VA/SCD−ve −4.4 ± 1.5 mV; *p* = .01).

To address the possibility that inclusion of small pedigrees might have underestimated the number of variants associated with a family history of SCD, a sensitivity analysis was performed including only variants identified in pedigrees with ≥5 individuals. In this subset, a higher prevalence of VA/SCD was seen (75/91 [82%]). This sensitivity analysis did not affect the relationship between VA/SCD and any aspect of channel function, and all relationships remained nonsignificant.

As the apparent effect of variants was found to vary between expression systems, we performed a further sensitivity analysis to remove this source of heterogeneity by including only variants expressed in HEK/TSA cells. This sensitivity analysis did not affect the relationship between CA, VA/SCD, or spontaneous ECG with any aspect of channel function, and all relationships remained nonsignificant.

## 4 DISCUSSION

The findings of this systematic analysis of functionally characterized *SCN5A* variants underlying BrS are as follows. First, the primary analysis showed no significant relationship between aspects of channel function and CA, VA or family history of SCD, or a history of a spontaneous type 1 ECG pattern. Second, sensitivity analyses suggested a weak relationship between decrease in peak *I*_Na_ and a positive shift in steady‐state activation with both CA and VA/SCD, although sensitivity and specificity remained low.

As can be seen from the beeswarm plots in Figures 1, 2, 3, the degree of channel perturbation was widely spread across all groups. Some variants such as the missense G35S are associated with a spontaneous BrS ECG and VA without any apparent effect on channel function (Gutter et al., 2013; Levy‐Nissenbaum et al., 2001). Conversely, some variants such as the missense variant I1660V cause marked abnormalities in channel function but were associated with only mild clinical phenotypes (Cordeiro et al., 2006; Selga et al., 2015).

Previous work has suggested that variants with ≥90% attenuation of *I*_Na_ are more likely to have a prolonged PR interval and present with syncope (Meregalli et al., 2009). Although we did not identify such a relationship in our primary analysis, two of our sensitivity analyses demonstrated a modest association between *I*_Na_ attenuation and CA, with optimal cutoffs for detection between 85% and 95% peak current reduction. In vitro variables had minimal ability to risk stratify families for VA. These findings contrast with previous reports indicating that the presence of an *SCN5A* variant confers an increased risk of VA (Chen et al., 2019), and that greater attenuation of peak *I*_Na_ and a more positive shift in steady‐state activation are associated with increased penetrance of BrS (Kroncke et al., 2018). We did not observe a relationship between the presence of a spontaneous BrS ECG and any aspect channel function in our primary or sensitivity analyses. We are unaware of previous work linking channel function with the presence of a spontaneous versus drug‐induced BrS ECG pattern, but a spontaneous BrS ECG is generally considered to be associated with a more malignant phenotype, and might, therefore, be predicted to be associated with a greater degree of channel perturbation (Priori et al., 2015), although we failed to find such a relationship.

There are a number of potential explanations for our observations. First, a robust correlation would only be expected if BrS were a monogenic disorder. However, increasing evidence indicates that BrS is an oligo‐ or polygenic disorder with complex inheritance (Bezzina et al., 2013; Probst et al., 2009). Within the same pedigree, some members carrying an *SCN5A* variant manifest a mild clinical phenotype, some a severe phenotype, while others have a BrS phenotype without carrying the familial variant (Probst et al., 2009). The findings presented here reinforce the hypothesis that the BrS phenotype is a product of multiple genetic influences (Cerrone et al., 2019). The impact of a single rare *SCN5A* variant is likely to be modulated by more common variants in related susceptibility genes, blurring the relationship between any single variant and phenotype (Cerrone et al., 2019). Second, the clinical effect of some variants may have been underestimated due to limited pedigree sizes, although the lack of association between functional parameters and arrhythmic risk remained when limiting analysis to larger pedigrees. Third, *SCN5A* variants may have additional effects beyond modulating *I*_Na_, including influencing potassium currents such as *I*_K1_ and affecting myocardial fibrosis (Jeevaratnam et al., 2016; Perez‐Hernandez et al., 2018). These may confer an arrhythmic phenotype without influencing the ion‐carrying aspects of sodium channel function explored here. There are multiple proposed mechanisms underlying the BrS ECG pattern. A widely held view is that an imbalance in repolarization between the epi‐ and endocardial layers of the right ventricular outflow tract due to *I*_Na_ attenuation underlies the BrS ECG. However, alternative proposed mechanisms relate to structural abnormalities of the outflow tract (Hoogendijk et al., 2010). These structural changes may still relate to variants in *SCN5A* but potentially due to noncanonical roles of the sodium channel in cell‐to‐cell adhesion, as suggested by studies of *SCN5A* variants implicated in arrhythmogenic cardiomyopathy (Te Riele et al., 2017). If these noncanonical roles are a key determinant of the BrS ECG pattern, the effects of *SCN5A* variants on *I*_Na_ may be less likely to associate with this aspect of phenotype.

### 4.1 Limitations

Significant heterogeneity was observed between studies in terms of the techniques used for functional analysis. In particular, there was significant variation in the expression systems used and coexpression of beta‐ and wild‐type subunits, which are known to influence the apparent effect of variants. Furthermore, a small minority used myocyte‐specific expression systems. These factors could potentially have had a significant impact on the observed genotype–phenotype correlations. However, these data reflect real‐world practice in which functional testing is recommended for use as evidence for variant pathogenicity by the ACMG (Richards et al., 2015), and guidelines specific to cardiac channelopathies (Campuzano et al., 2015). Importantly, these scoring systems do not give guidance as to the experimental conditions to be used or even to the degree of channel dysfunction needed to denote pathogenicity. Overall, our findings highlight the importance of a standardized approach to sodium channel assessment.

Clinical phenotyping is presented here in a binary form owing to the limitations of the source material. Ideally, the prevalence of phenotypes such as SCD would be expressed as continuous variables. More specifically, using large pedigrees, the prevalence of SCD would be expressed as a proportion of the total genotype‐positive pedigree. However, owing to a lack of sufficient detail in many of the included manuscripts, this approach was not feasible. While variants were excluded for which clinical data were solely available for compound mutations, it is possible that unrecognized compound mutations in nongenotyped family members could have been responsible for SCD, potentially overestimating pathogenic effects. Details of age and gender, factors known to influence the BrS phenotype, were not available for many variants and these potential confounders have not been included in the analysis. Publication bias in favor of reporting variants associated with more malignant presentations may have biased the dataset.

Associations seen only in sensitivity analyses should be viewed as hypothesis generating, as apparent statistical significance may have been biased by multiple‐hypothesis testing.

### 4.2 Implications and future work

The evidence presented here does not support in vitro functional assessment of *SCN5A* variants to refine risk stratification. Furthermore, it raises the question whether scoring systems of pathogenicity should place such strong evidence on functional assessment.

We encourage further studies to adopt a standardized approach to sodium channel assessment. Future studies should incorporate factors shown to influence the apparent effects of *SCN5A* variants including use of a mammalian expression system (Baroudi et al., 2001), coexpression of beta subunits (Makita et al., 2000), and recording currents at physiological temperatures (Amin et al., 2005). Furthermore, variants should be expressed heterozygously alongside wild‐type subunits allowing any dominant negative effects to be taken into account (Hoshi et al., 2014). Currents should be characterized in full including current/voltage relationships, steady‐state activation and inactivation, current decay, and recovery from inactivation fitted to single or double exponentials. We suggest that that minor derangement (e.g., <50% current reduction during homozygous expression) should not be regarded as evidence of pathogenicity due to the very low sensitivities seen in ROC analysis for VA/SCD (19%) or CA (31%) below these values.

Variant classification may be improved further by using induced human pluripotent stem cells (iPSCs). These models reflect overall gene expression profiles better than noncardiomyocyte cell lines which may influence the apparent effects of variants in unforeseen ways (Ross et al., 2018). By taking into account polymorphisms in non‐*SCN5A* genes thereby reflecting the oligogenic nature of BrS, iPSCs also offer the opportunity of patient‐specific variant characterization (Ross et al., 2018).

## 5 CONCLUSION

Probing the genotype–phenotype relationship in BrS based on current in vitro functional testing has shown that there is little relationship between sodium channel function and clinical phenotype. To better detect such a relationship, further in vitro studies should aim to replicate physiological conditions in their expression systems as closely as practicable including heterozygous expression, coexpression of beta subunits, and assessment using iPSCs, while further clinical studies should report corresponding phenotypes of all gene‐positive and gene‐negative family members in as large a pedigree as can be achieved. The current weight placed on functional testing for ascertainment of variant pathogenicity should be questioned, particularly when interpreting minor degrees of channel dysfunction.

## CONFLICT OF INTERESTS

The authors declare that there are no conflict of interests.

## Supporting information

Supporting information.

Supporting information.

## Data Availability

The full dataset which supports the findings of this study is available in the Supporting Infomation and from the corresponding author upon reasonable request.
