# Supplementary material for: Relationship between sodium channel function and clinical phenotype in SCN5A variants associated with Brugada syndrome
Source: Hum Mutat. 2020 Nov 11;41(12):2195–204. doi: 10.1002/humu.24128 (PMC7756571; doi:10.1002/humu.24128)
Supplement: Supplementary file 1 — Supporting information. [file HUMU-41-2195-s001.docx]

**Supplemental materials – full list of SCN5A variants associated with BrS**

| Variant type | Variant | Alternative name | | | Position | | ACMG score | | Spontaneous ventricular arrhythmias | | FHx SCD | | Ventricular Arrhythmias or FHx of SCD | | Spontaneous Type 1 ECG | | Conduction Abnormalities | | Sinus node dysfunction | | Any AV block | | High grade AV block | | Interventricular conduction delay | | QT prolongation | | Peak current (homozygous expression) | | Peak current (heterozygous expression) | | Current decay | | Steady state activation | | Steady state inactivation | Recovery from inactivation |
| --- | --- | --- | --- | --- | --- | --- | --- | --- | --- | --- | --- | --- | --- | --- | --- | --- | --- | --- | --- | --- | --- | --- | --- | --- | --- | --- | --- | --- | --- | --- | --- | --- | --- | --- | --- | --- | --- | --- |
| Non-coding / Splice | c.-53+1G>A |  | | | -17 | | VUS | |  | |  | |  | |  | |  | |  | |  | |  | |  | |  | |  | |  | |  | |  | |  |  |
| Missense | M1I |  | | | 1 | | VUS | |  | |  | |  | |  | |  | |  | |  | |  | |  | |  | |  | |  | |  | |  | |  |  |
| Missense | A2T |  | | | 2 | | VUS | | - | | - | | - | | - | | - | | - | | - | | - | | - | |  | |  | |  | |  | |  | |  |  |
| Premature truncation | M11X |  | | | 11 | | LP | |  | |  | |  | |  | | - | |  | |  | |  | |  | |  | |  | |  | |  | |  | |  |  |
| Missense | R18Q |  | | | 18 | | B | | + | | - | | + | | - | | - | | - | | - | | - | | - | | + | | 1 | |  | |  | | 0 | | 0 |  |
| Missense | R27H |  | | | 27 | | VUS | | - | | - | | - | | - | | - | | - | | - | | - | | - | |  | | 1 | |  | | No effect | | 4 | | 0 | No effect |
| Missense | G35S |  | | | 35 | | B | | + | | + | | + | | + | | - | | - | | - | | - | | - | |  | | 1 | |  | | No effect | | 0 | | 0 | No effect |
| Premature truncation | R43X |  | | | 43 | | LP | | - | | - | |  | |  | | - | |  | |  | |  | |  | |  | |  | |  | |  | |  | |  |  |
| Premature truncation | Q55X |  | | | 55 | | P | | - | | - | | - | | - | | + | | + | | + | | - | | - | |  | | 0 | |  | |  | |  | |  |  |
| Deletion | L64del |  | | | 64 | | VUS | |  | |  | |  | |  | | - | |  | |  | |  | |  | |  | |  | |  | |  | |  | |  |  |
| Missense | N70K |  | | | 70 | | VUS | |  | |  | |  | |  | | - | |  | |  | |  | |  | |  | | 0 | | 0.7 | | No effect | |  | | 0 |  |
| Premature truncation | Q73X |  | | | 73 | | LP | |  | |  | |  | |  | | - | |  | |  | |  | |  | |  | |  | |  | |  | |  | |  |  |
| Missense | D84N |  | | | 84 | | VUS | |  | |  | |  | |  | | - | |  | |  | |  | |  | |  | | 0.35 | |  | | Slower | | 8 | |  |  |
| Missense | Y87C |  | | | 87 | | VUS | |  | |  | |  | |  | |  | |  | |  | |  | |  | |  | |  | |  | |  | |  | |  |  |
| Non-coding / Splice | c.274-24C>T |  | | | 92 | | VUS | |  | |  | |  | |  | |  | |  | |  | |  | |  | |  | |  | |  | |  | |  | |  |  |
| Missense | F93S |  | | | 93 | | VUS | |  | |  | |  | |  | |  | |  | |  | |  | |  | |  | | 0 | |  | |  | |  | |  |  |
| Missense | I94S |  | | | 94 | | VUS | |  | |  | |  | |  | | - | |  | |  | |  | |  | |  | |  | |  | |  | |  | |  |  |
| Missense | V95I |  | | | 95 | | VUS | | - | | + | | + | | + | | - | | - | | - | | - | | - | |  | | 1 | |  | | No effect | | 0 | | 0 | No effect |
| Missense | R104Q |  | | | 104 | | LP | | + | | + | | + | | + | | - | | - | | - | | - | | - | |  | | 0 | |  | | No effect | | 0 | | 0 | No effect |
| Missense | R104W |  | | | 104 | | LP | | + | | + | | + | | + | | - | | - | | - | | - | | - | |  | | 0 | | 0.2 | |  | |  | |  |  |
| Missense | N109K |  | | | 109 | | VUS | |  | |  | |  | | - | | - | |  | |  | |  | |  | |  | | 1.2 | |  | | No effect | | -1 | | 1 | No effect |
| Missense | R121W |  | | | 121 | | LP | | + | | - | | + | | + | | + | | + | | + | | + | | + | |  | | 0 | | 0.2 | |  | |  | |  |  |
| Missense | R121Q |  | | | 121 | | VUS | |  | |  | |  | |  | | - | |  | |  | |  | |  | |  | |  | |  | |  | |  | |  |  |
| Missense | A124D |  | | | 124 | | LP | | - | | - | | - | | + | | - | | - | | - | | - | | - | |  | | 0.2 | |  | |  | | 0 | | 0 |  |
| Missense | K126E |  | | | 126 | | VUS | |  | |  | |  | |  | | - | |  | |  | |  | |  | |  | | 1 | |  | | No effect | | 3 | | 4 | No effect |
| Frameshift | L128SfsX44 | c.381dupT | | | 128 | | LP | |  | |  | |  | |  | | - | |  | |  | |  | |  | |  | |  | |  | |  | |  | |  |  |
| Non-coding / Splice | c.393-5C>A |  | | | 132 | | VUS | |  | |  | |  | | - | | - | |  | |  | |  | |  | |  | |  | |  | |  | |  | |  |  |
| Non-coding / Splice | c.393-1C>T |  | | | 132 | | VUS | | + | | - | |  | | - | | - | |  | |  | |  | |  | |  | |  | |  | |  | |  | |  |  |
| Missense | L136P |  | | | 136 | | VUS | | - | | - | | - | |  | | - | | - | | - | | - | | - | |  | | 0.4 | |  | | Slower | |  | |  |  |
| Duplication | I137_C139dup |  | | | 137 | | VUS | |  | |  | |  | |  | | - | |  | |  | |  | |  | |  | |  | |  | |  | |  | |  |  |
| Missense | V146M |  | | | 146 | | VUS | |  | |  | |  | |  | | - | |  | |  | |  | |  | |  | |  | |  | |  | |  | |  |  |
| Premature truncation | W156X |  | | | 156 | | P | | + | | - | | + | | - | | + | | - | | + | | + | | + | |  | | 0 | |  | |  | |  | |  |  |
| Premature truncation | Y159X |  | | | 159 | | LP | | - | | - | |  | |  | | - | |  | |  | |  | |  | |  | |  | |  | |  | |  | |  |  |
| Frameshift | V160fs |  | | | 160 | | VUS | |  | |  | |  | |  | |  | |  | |  | |  | |  | |  | |  | |  | |  | |  | |  |  |
| Missense | E161K |  | | | 161 | | VUS | | - | | - | | - | | + | | + | | + | | + | | - | | + | |  | | 0.4 | |  | | No effect | | 12 | | 0 | No effect |
| Missense | E161Q |  | | | 161 | | VUS | |  | |  | |  | |  | | - | |  | |  | |  | |  | |  | |  | |  | |  | |  | |  |  |
| Frameshift | Y162XfsX1 | c.486delC | | | 162 | | LP | |  | |  | |  | |  | | - | |  | |  | |  | |  | |  | |  | |  | |  | |  | |  |  |
| Missense | K175N |  | | | 176 | | VUS | |  | |  | |  | |  | | - | |  | |  | |  | |  | |  | | 1.2 | |  | | No effect | | 7 | | -9 |  |
| Missense | A178G |  | | | 178 | | VUS | |  | |  | |  | |  | | - | |  | |  | |  | |  | |  | | 1.1 | |  | | No effect | | 9 | |  |  |
| Premature truncation | R179X |  | | | 179 | | P | | + | | - | | + | | + | | - | | - | | - | | - | | - | |  | | 0 | |  | |  | |  | |  |  |
| Missense | G180V |  | | | 180 | | VUS | |  | |  | |  | |  | |  | |  | |  | |  | |  | |  | |  | |  | |  | |  | |  |  |
| Missense | C182R |  | | | 182 | | VUS | |  | |  | |  | |  | | - | |  | |  | |  | |  | |  | |  | |  | |  | |  | |  |  |
| Missense | A185V |  | | | 185 | | VUS | |  | |  | |  | |  | | - | |  | |  | |  | |  | |  | |  | |  | |  | |  | |  |  |
| Missense | T187I |  | | | 187 | | LP | | + | | - | | + | | + | | + | | + | | - | | - | | - | |  | | 0 | | 0.5 | |  | | 0 | | 0 |  |
| Missense | R190Q |  | | | 190 | | VUS | |  | |  | |  | |  | |  | |  | |  | |  | |  | |  | |  | |  | |  | |  | |  |  |
| Premature truncation | W193X | c.611 C>T | | | 193 | | LP | |  | |  | |  | |  | | - | |  | |  | |  | |  | |  | |  | |  | |  | |  | |  |  |
| Missense | A204V |  | | | 204 | | VUS | |  | |  | |  | |  | | - | |  | |  | |  | |  | |  | |  | |  | |  | |  | |  |  |
| Non-coding / Splice | c.611+1G>A |  | | | 204 | | VUS | |  | |  | |  | |  | | - | |  | |  | |  | |  | |  | |  | |  | |  | |  | |  |  |
| Non-coding / Splice | c.611+3_611+4dupAA | 204 | VUS |  | |  | |  | |  | | - | |  | |  | |  | |  | |  | |  | |  | |  | |  | |  | |  | |  |  |  |
| Non-coding / Splice | c.612-2A>G |  | | | 205 | | VUS | | - | | + | |  | | + | | + | |  | |  | |  | |  | |  | |  | |  | |  | |  | |  |  |
| Missense | L212Q |  | | | 212 | | VUS | |  | |  | |  | |  | | - | |  | |  | |  | |  | |  | |  | |  | |  | |  | |  |  |
| Missense | S216L |  | | | 216 | | VUS | | + | | + | | + | | + | | - | | - | | - | | - | | - | | + | | 0.4 | |  | |  | | 0 | | 0 | Slower |
| Frameshift | T220FfsX10 | c.656_657insATTCA | | | 220 | | LP | |  | |  | |  | |  | | - | |  | |  | |  | |  | |  | |  | |  | |  | |  | |  |  |
| Missense | T220I |  | | | 220 | | VUS | |  | |  | |  | | - | | - | |  | |  | |  | |  | |  | | 0.85 | |  | | No effect | | -3 | | -4 | Slower |
| Premature truncation | R222X |  | | | 222 | | P | | - | | + | | + | | + | | + | | - | | - | | - | | - | |  | | 0 | |  | |  | |  | |  |  |
| Missense | R222Q |  | | | 222 | | VUS | | + | | - | | + | |  | | + | | - | | + | | + | | - | | + | | 1 | | 1 | | Faster | | -17 | | -13 |  |
| Missense | V223L |  | | | 223 | | VUS | |  | |  | |  | |  | | - | |  | |  | |  | |  | |  | | 0.35 | |  | | No effect | | -2 | | -3 |  |
| Missense | R225W |  | | | 225 | | VUS | | - | | - | | - | | + | | + | | - | | + | | - | | - | |  | | 0.1 | |  | |  | | 18 | | 10 | No effect |
| Missense | A226V |  | | | 226 | | VUS | | - | | - | | - | | - | | - | | - | | - | | - | | - | |  | | 0.25 | |  | |  | | 0 | | 0 |  |
| Missense | L227P |  | | | 227 | | VUS | |  | |  | |  | |  | |  | |  | |  | |  | |  | |  | |  | |  | |  | |  | |  |  |
| Missense | I230V |  | | | 230 | | VUS | |  | |  | |  | |  | | - | |  | |  | |  | |  | |  | |  | |  | |  | |  | |  |  |
| Frameshift | c.692_693delCA |  | | | 231 | | VUS | |  | |  | |  | |  | |  | |  | |  | |  | |  | |  | |  | |  | |  | |  | |  |  |
| Missense | V232I |  | | | 232 | | VUS | |  | |  | |  | | - | | - | |  | |  | |  | |  | |  | |  | |  | |  | |  | |  |  |
| Non-coding / Splice | c.704-1G>C |  | | | 235 | | VUS | |  | |  | |  | |  | |  | |  | |  | |  | |  | |  | |  | |  | |  | |  | |  |  |
| Missense | V240M |  | | | 240 | | VUS | |  | |  | |  | | - | | - | |  | |  | |  | |  | |  | |  | |  | |  | |  | |  |  |
| Premature truncation | K249X |  | | | 249 | | LP | |  | |  | |  | |  | | - | |  | |  | |  | |  | |  | |  | |  | |  | |  | |  |  |
| Missense | Q270K |  | | | 270 | | VUS | | + | | - | |  | | - | | + | |  | |  | |  | |  | |  | |  | |  | |  | |  | |  |  |
| Missense | L276Q |  | | | 276 | | VUS | | + | | - | | + | | + | | - | | - | | - | | - | | - | |  | | 0 | |  | |  | |  | |  |  |
| Missense | L276P |  | | | 276 | | VUS | |  | |  | |  | |  | |  | |  | |  | |  | |  | |  | |  | |  | |  | |  | |  |  |
| Missense | H278D |  | | | 278 | | VUS | |  | |  | |  | |  | | - | |  | |  | |  | |  | |  | |  | |  | |  | |  | |  |  |
| Missense | H278R |  | | | 278 | | VUS | |  | |  | |  | |  | | - | |  | |  | |  | |  | |  | |  | |  | |  | |  | |  |  |
| Missense | C280Y |  | | | 280 | | VUS | |  | |  | |  | |  | | - | |  | |  | |  | |  | |  | |  | |  | |  | |  | |  |  |
| Missense | V281M |  | | | 281 | | VUS | |  | |  | |  | | - | | - | |  | |  | |  | |  | |  | |  | |  | |  | |  | |  |  |
| Missense | R282H |  | | | 282 | | LP | | + | | + | | + | | + | | + | | - | | - | | - | | + | |  | | 0.1 | | 0.6 | |  | | 5 | | 5 | No effect |
| Missense | R282C |  | | | 282 | | VUS | |  | |  | |  | |  | | - | |  | |  | |  | |  | |  | | 0 | |  | | Slower | |  | |  |  |
| Non-coding / Splice | c.851insTG |  | | | 284 | | VUS | | + | | + | |  | | + | | + | |  | |  | |  | |  | |  | |  | |  | |  | |  | |  |  |
| Frameshift | c.870delC |  | | | 291 | | VUS | |  | |  | |  | |  | |  | |  | |  | |  | |  | |  | |  | |  | |  | |  | |  |  |
| Missense | G292S |  | | | 292 | | VUS | | - | | + | |  | | - | | - | |  | |  | |  | |  | |  | |  | |  | |  | |  | |  |  |
| Missense | V294M |  | | | 294 | | VUS | |  | |  | |  | | - | | - | |  | |  | |  | |  | |  | |  | |  | |  | |  | |  |  |
| Missense | V300I |  | | | 300 | | VUS | |  | |  | |  | |  | | - | |  | |  | |  | |  | |  | |  | |  | |  | |  | |  |  |
| Premature truncation | T301X |  | | | 301 | | LP | | + | | + | | + | | + | | - | | - | | - | | - | | - | |  | |  | |  | |  | |  | |  |  |
| Missense | E312K |  | | | 312 | | VUS | |  | |  | |  | |  | | - | |  | |  | |  | |  | |  | |  | |  | |  | |  | |  |  |
| Non-coding / Splice | c.934+1G>A |  | | | 312 | | VUS | |  | |  | |  | | - | | - | |  | |  | |  | |  | |  | |  | |  | |  | |  | |  |  |
| Non-coding / Splice | c.934+4C>T |  | | | 312 | | VUS | |  | |  | |  | |  | | - | |  | |  | |  | |  | |  | |  | |  | |  | |  | |  |  |
| Missense | L315P |  | | | 315 | | VUS | |  | |  | |  | |  | | - | |  | |  | |  | |  | |  | |  | |  | |  | |  | |  |  |
| Missense | K317N |  | | | 317 | | VUS | |  | |  | |  | |  | |  | |  | |  | |  | |  | |  | |  | |  | |  | |  | |  |  |
| Missense | G319S |  | | | 319 | | VUS | | - | | - | |  | | - | | - | |  | |  | |  | |  | |  | |  | |  | |  | |  | |  |  |
| Missense | T320N |  | | | 320 | | VUS | |  | |  | |  | |  | | - | |  | |  | |  | |  | |  | |  | |  | |  | |  | |  |  |
| Missense | S321Y |  | | | 321 | | VUS | | - | | - | |  | | + | | - | |  | |  | |  | |  | |  | |  | |  | |  | |  | |  |  |
| Missense | L325R |  | | | 325 | | LP | | + | | - | | + | | + | | - | | - | | - | | - | | - | |  | | 0.2 | | 0.25 | | Slower | | 10 | | 0 |  |
| Non-coding / Splice | c.998+1G>A |  | | | 333 | | VUS | |  | |  | |  | |  | | - | |  | |  | |  | |  | |  | |  | |  | |  | |  | |  |  |
| Non-coding / Splice | c.999-424_1338+81del | 334 | VUS | - | | - | |  | | + | | + | |  | |  | |  | |  | |  | |  | |  | |  | |  | |  | |  | |  |  |  |
| Missense | C335S |  | | | 335 | | VUS | |  | |  | |  | |  | | - | |  | |  | |  | |  | |  | |  | |  | |  | |  | |  |  |
| Missense | C335R |  | | | 335 | | VUS | | - | | + | | + | | + | | + | | + | | - | | - | | - | |  | | 0 | |  | |  | |  | |  |  |
| Missense | P336L |  | | | 336 | | LP | | - | | - | | - | | + | | + | | - | | + | | - | | - | |  | | 0.15 | |  | |  | | 0 | | 0 |  |
| Premature truncation | E346X |  | | | 346 | | P | | - | | - | | - | |  | | - | | - | | - | | - | | - | |  | | 0 | |  | |  | |  | |  |  |
| Missense | E346D |  | | | 346 | | VUS | |  | |  | |  | |  | |  | |  | |  | |  | |  | |  | | 1.15 | |  | | No effect | | -8 | | -4 | Slower |
| Missense | D349N |  | | | 349 | | LP | |  | |  | |  | |  | |  | |  | |  | |  | |  | |  | | 0.3 | |  | |  | | 10 | |  |  |
| Missense | G351V |  | | | 351 | | LP | | - | | + | | + | |  | | - | | - | | - | | - | | - | |  | | 0.15 | |  | | Slower | | 0 | | 0 | No effect |
| Missense | G351D |  | | | 351 | | VUS | |  | |  | |  | |  | | - | |  | |  | |  | |  | |  | |  | |  | |  | |  | |  |  |
| Missense | Y352C |  | | | 352 | | VUS | | + | | + | | + | | + | | - | | - | | - | | - | | - | |  | | 0.15 | | 0.5 | |  | | 0 | | -10 |  |
| Missense | T353I |  | | | 353 | | LP | | + | | + | | + | | + | | + | | - | | + | | - | | - | | + | | 0 | |  | | Faster | | -1 | | -13 |  |
| Missense | F355C |  | | | 355 | | VUS | |  | |  | |  | |  | |  | |  | |  | |  | |  | |  | |  | |  | |  | |  | |  |  |
| Missense | D356N |  | | | 356 | | VUS | | + | | - | | + | | + | | + | | - | | + | | + | | - | |  | | 0 | |  | |  | | 0 | | 0 |  |
| Missense | R367C |  | | | 367 | | LP | | + | | + | | + | | - | | - | | - | | - | | - | | - | | + | | 0 | |  | |  | |  | |  |  |
| Missense | R367H |  | | | 367 | | VUS | | + | | - | | + | | + | | + | | + | | - | | - | | - | | + | | 0 | | 0.5 | |  | | 8 | | -9 |  |
| Missense | R367L |  | | | 367 | | VUS | |  | |  | |  | |  | | - | |  | |  | |  | |  | |  | | 0 | |  | |  | |  | |  |  |
| Missense | M369K |  | | | 369 | | LP | | - | | - | |  | | + | | - | |  | |  | |  | |  | |  | | 0.05 | |  | |  | |  | |  |  |
| Premature truncation | Q371X |  | | | 371 | | LP | | + | | + | | + | | - | | + | | + | | - | | - | | - | |  | |  | |  | |  | |  | |  |  |
| Missense | W374G |  | | | 374 | | VUS | |  | |  | |  | |  | | - | |  | |  | |  | |  | |  | |  | |  | |  | |  | |  |  |
| Missense | R376H |  | | | 376 | | LP | | + | | + | | + | | + | | + | | + | | + | | - | | + | |  | | 0.05 | |  | |  | | 0 | | 0 |  |
| Non-coding / Splice | c.1140+1G>A |  | | | 381 | | VUS | |  | |  | |  | |  | |  | |  | |  | |  | |  | |  | |  | |  | |  | |  | |  |  |
| Non-coding / Splice | c.1141-3C>A |  | | | 381 | | VUS | |  | |  | |  | |  | |  | |  | |  | |  | |  | |  | |  | |  | |  | |  | |  |  |
| Missense | G386R |  | | | 386 | | VUS | | - | | - | | - | | + | | - | | - | | - | | - | | - | |  | | 0 | |  | |  | |  | |  |  |
| Missense | G386E |  | | | 386 | | VUS | |  | |  | |  | |  | | - | |  | |  | |  | |  | |  | |  | |  | |  | |  | |  |  |
| Deletion | F393del |  | | | 393 | | VUS | |  | |  | |  | |  | | - | |  | |  | |  | |  | |  | |  | |  | |  | |  | |  |  |
| Missense | V396L |  | | | 396 | | VUS | |  | |  | |  | |  | | - | |  | |  | |  | |  | |  | | 0.3 | |  | | Slower | | 3 | | 1 | Faster |
| Missense | V396A |  | | | 396 | | VUS | |  | |  | |  | |  | | - | |  | |  | |  | |  | |  | |  | |  | |  | |  | |  |  |
| Missense | I397V |  | | | 397 | | VUS | |  | |  | |  | |  | | - | |  | |  | |  | |  | |  | |  | |  | |  | |  | |  |  |
| Missense | G400R |  | | | 400 | | VUS | |  | |  | |  | |  | |  | |  | |  | |  | |  | |  | |  | |  | |  | |  | |  |  |
| Missense | N406S |  | | | 406 | | VUS | | + | | + | | + | | + | | + | | - | | + | | - | | + | |  | | 1 | |  | | Faster | | 15 | | 10 | Faster |
| Missense | Y416C |  | | | 416 | | VUS | | - | |  | |  | |  | |  | |  | |  | |  | |  | |  | |  | |  | |  | |  | |  |  |
| Premature truncation | Q419X |  | | | 419 | | LP | |  | |  | |  | |  | | - | |  | |  | |  | |  | |  | |  | |  | |  | |  | |  |  |
| Missense | E428K |  | | | 428 | | VUS | | - | |  | | - | |  | |  | |  | |  | |  | |  | |  | | 1.5 | |  | |  | | 0 | | 0 | No effect |
| Missense | E439K |  | | | 439 | | LP | |  | |  | |  | | - | | - | |  | |  | |  | |  | |  | | 0 | | 0.4 | |  | |  | | 0 |  |
| Missense | H445D |  | | | 445 | | VUS | |  | |  | |  | |  | | - | |  | |  | |  | |  | |  | |  | |  | |  | |  | |  |  |
| Non-coding / Splice | c.1338+2T>A |  | | | 447 | | VUS | |  | |  | |  | |  | | - | |  | |  | |  | |  | |  | |  | |  | |  | |  | |  |  |
| Missense | L466F |  | | | 466 | | VUS | |  | |  | |  | |  | |  | |  | |  | |  | |  | |  | |  | |  | |  | |  | |  |  |
| Missense | P468L |  | | | 468 | | LB | | + | | - | | + | | + | | - | | - | | - | | - | | - | |  | | 1 | |  | | No effect | | 0 | | 0 |  |
| Premature truncation | E473X |  | | | 473 | | P | | - | | - | | - | | + | | - | | - | | - | | - | | - | |  | | 0 | | 0.5 | |  | |  | |  |  |
| Missense | R475K |  | | | 475 | | VUS | |  | |  | |  | |  | | - | |  | |  | |  | |  | |  | |  | |  | |  | |  | |  |  |
| Frameshift | S476RfsX30 | c.1428_1431delCAAG | | | 476 | | LP | |  | |  | |  | |  | | - | |  | |  | |  | |  | |  | |  | |  | |  | |  | |  |  |
| Missense | R481W |  | | | 481 | | VUS | |  | |  | |  | |  | |  | |  | |  | |  | |  | |  | |  | |  | |  | |  | |  |  |
| Deletion | K493 (1479delK) |  | | | 493 | | VUS | |  | |  | |  | |  | | - | |  | |  | |  | |  | |  | |  | |  | |  | |  | |  |  |
| Missense | D501G |  | | | 501 | | VUS | |  | |  | |  | |  | | - | |  | |  | |  | |  | |  | |  | |  | |  | |  | |  |  |
| Missense | T512I |  | | | 512 | | VUS | |  | |  | |  | |  | |  | |  | |  | |  | |  | |  | |  | |  | |  | |  | |  |  |
| Frameshift | R513VfsX8 | c.1537delC | | | 513 | | LP | |  | |  | |  | |  | | - | |  | |  | |  | |  | |  | |  | |  | |  | |  | |  |  |
| Missense | R513H |  | | | 513 | | VUS | |  | |  | |  | |  | | - | |  | |  | |  | |  | |  | |  | |  | |  | |  | |  |  |
| Missense | G514C |  | | | 514 | | VUS | |  | | + | | + | |  | | + | | + | | - | | - | | + | |  | | 1 | |  | | Faster | | 10 | | 7 | No effect |
| Missense | S519F |  | | | 519 | | VUS | | - | | - | |  | | + | | - | |  | |  | |  | |  | |  | |  | |  | |  | |  | |  |  |
| Frameshift | K521SfsX102 | c.1562delA | | | 521 | | LP | |  | |  | |  | |  | | - | |  | |  | |  | |  | |  | |  | |  | |  | |  | |  |  |
| Frameshift | c.1570_1571insG |  | | | 524 | | VUS | |  | |  | |  | |  | |  | |  | |  | |  | |  | |  | |  | |  | |  | |  | |  |  |
| Missense | R526H |  | | | 526 | | VUS | | - | | + | | + | | + | | - | | - | | - | | - | | - | |  | | 0.7 | | 1 | |  | | 0 | | 0 | No effect |
| Missense | F532C |  | | | 532 | | VUS | | - | | - | |  | |  | | - | |  | |  | |  | |  | |  | |  | |  | |  | |  | |  |  |
| Premature truncation | R535X |  | | | 535 | | P | | + | | + | | + | | + | | + | | - | | - | | - | | - | |  | | 0 | |  | |  | |  | |  |  |
| Missense | F543L |  | | | 543 | | VUS | |  | |  | |  | |  | | - | |  | |  | |  | |  | |  | |  | |  | |  | |  | |  |  |
| Missense | A551T |  | | | 551 | | LP | | + | | + | | + | | + | | + | | - | | + | | - | | + | |  | | 0.5 | |  | | No effect | | 4 | | -3 | Slower |
| Missense | G552R |  | | | 552 | | VUS | |  | |  | |  | |  | | - | |  | |  | |  | |  | |  | | 0 | | 0.5 | |  | |  | | 0 |  |
| Premature truncation | E553X |  | | | 553 | | LP | |  | |  | |  | |  | |  | |  | |  | |  | |  | |  | |  | |  | |  | |  | |  |  |
| Missense | E555K |  | | | 555 | | VUS | | - | | + | | + | | + | | - | | - | | - | | - | | - | |  | |  | | 0.7 | |  | |  | | 0 |  |
| Missense | L567Q |  | | | 567 | | VUS | | + | | + | | + | | - | | - | | - | | - | | - | | - | |  | | 0.65 | | 0.35 | | Faster | | 6 | | -11 | No effect |
| Frameshift | R569Pfs151 |  | | | 569 | | LP | | - | | - | |  | | - | | - | |  | |  | |  | |  | |  | |  | |  | |  | |  | |  |  |
| Frameshift | c.1705dupC |  | | | 569 | | VUS | |  | |  | |  | |  | |  | |  | |  | |  | |  | |  | |  | |  | |  | |  | |  |  |
| Missense | A572D |  | | | 572 | | VUS | |  | |  | |  | |  | |  | |  | |  | |  | |  | |  | |  | |  | |  | |  | |  |  |
| Premature truncation | Q573X |  | | | 573 | | LP | | - | | + | |  | | + | | - | |  | |  | |  | |  | |  | |  | |  | |  | |  | |  |  |
| Frameshift | G574DfsX49 | c.1721delG | | | 574 | | LP | |  | |  | |  | |  | | - | |  | |  | |  | |  | |  | |  | |  | |  | |  | |  |  |
| Missense | A586T |  | | | 586 | | VUS | | - | | - | | - | | - | | - | | - | | - | | - | | - | |  | |  | |  | |  | |  | |  |  |
| Missense | N592K |  | | | 592 | | VUS | | + | | + | | + | | + | | + | | - | | - | | - | | + | |  | | 0.15 | |  | |  | | 3 | | 1 |  |
| Missense | G615E |  | | | 615 | | VUS | |  | |  | |  | |  | | - | |  | |  | |  | |  | |  | | 1 | |  | | Faster | | 0 | |  |  |
| Missense | L618F |  | | | 618 | | VUS | |  | |  | |  | |  | |  | |  | |  | |  | |  | |  | |  | |  | |  | |  | |  |  |
| Missense | L619F |  | | | 619 | | VUS | |  | |  | |  | |  | | - | |  | |  | |  | |  | |  | |  | |  | |  | |  | |  |  |
| Missense | R620C |  | | | 620 | | VUS | |  | |  | |  | |  | | - | |  | |  | |  | |  | |  | | 0 | | 1 | |  | |  | | 0 |  |
| Frameshift | E625Rfs95 |  | | | 625 | | LP | | - | | + | |  | | + | | - | |  | |  | |  | |  | |  | |  | |  | |  | |  | |  |  |
| Frameshift | c.1872dupA |  | | | 625 | | VUS | |  | |  | |  | |  | |  | |  | |  | |  | |  | |  | |  | |  | |  | |  | |  |  |
| Non-coding / Splice | T630T | c.1890G>A | | | 630 | | VUS | |  | |  | |  | |  | |  | |  | |  | |  | |  | |  | |  | |  | |  | |  | |  |  |
| Non-coding / Splice | c.1890+5G>A |  | | | 631 | | VUS | |  | |  | |  | |  | | - | |  | |  | |  | |  | |  | |  | |  | |  | |  | |  |  |
| Missense | T632M |  | | | 632 | | VUS | |  | |  | |  | |  | | - | |  | |  | |  | |  | |  | | 0 | | 1 | |  | |  | | -2 |  |
| Missense | P640A |  | | | 640 | | VUS | |  | |  | |  | |  | | - | |  | |  | |  | |  | |  | |  | |  | |  | |  | |  |  |
| Frameshift | Q646Rfs+X5 | c.1936delC | | | 646 | | VUS | | + | | + | |  | | + | | + | |  | |  | |  | |  | |  | |  | |  | |  | |  | |  |  |
| Missense | A647D |  | | | 647 | | VUS | | - | | - | | - | |  | | - | | - | | - | | - | | - | |  | | 0 | | 0.4 | |  | |  | | 0 |  |
| Missense | P648L |  | | | 648 | | LB | | + | | - | | + | |  | | - | | - | | - | | - | | - | | + | | 1 | |  | |  | | -4 | | 0 | Slower |
| Frameshift | D651AfsX25 | c.1950_1953delAGAT | | | 651 | | LP | |  | |  | |  | |  | | - | |  | |  | |  | |  | |  | |  | |  | |  | |  | |  |  |
| Missense | R661W |  | | | 661 | | VUS | |  | |  | |  | |  | | - | |  | |  | |  | |  | |  | |  | |  | |  | |  | |  |  |
| Frameshift | A665fsX16 | c.1983_1993dupGGCCCTCAGCG | | | 665 | | VUS | |  | |  | |  | | - | | - | |  | |  | |  | |  | |  | |  | |  | |  | |  | |  |  |
| Frameshift | E675VfsX45 | c.2024_2025delAG | | | 675 | | LP | |  | |  | |  | |  | | - | |  | |  | |  | |  | |  | |  | |  | |  | |  | |  |  |
| Missense | H681P |  | | | 681 | | VUS | | - | | - | | - | | + | | - | | - | | - | | - | | - | |  | | 1 | |  | |  | | -10 | | -17 |  |
| Missense | C683G |  | | | 683 | | VUS | |  | |  | |  | |  | | - | |  | |  | |  | |  | |  | |  | |  | |  | |  | |  |  |
| Missense | R689H |  | | | 689 | | VUS | | - | | + | | + | | + | | - | | - | | - | | - | | - | | SQT | | 0 | |  | |  | |  | |  |  |
| Missense | R693C |  | | | 693 | | VUS | | + | |  | |  | |  | |  | |  | |  | |  | |  | |  | |  | |  | |  | |  | |  |  |
| Premature truncation | W697X |  | | | 697 | | LP | | - | | - | | - | | + | | - | | - | | - | | - | | - | |  | |  | |  | |  | |  | |  |  |
| Premature truncation | E698X |  | | | 698 | | LP | |  | |  | |  | |  | | - | |  | |  | |  | |  | |  | |  | |  | |  | |  | |  |  |
| Missense | P701L |  | | | 701 | | VUS | |  | |  | |  | |  | | - | |  | |  | |  | |  | |  | |  | | 1 | |  | |  | | 0 |  |
| Missense | M704T |  | | | 704 | | VUS | |  | |  | |  | |  | | - | |  | |  | |  | |  | |  | |  | |  | |  | |  | |  |  |
| Missense | P717L |  | | | 717 | | VUS | |  | |  | |  | |  | | - | |  | |  | |  | |  | |  | |  | |  | |  | |  | |  |  |
| Missense | V728I |  | | | 728 | | VUS | | - | | + | |  | | + | | - | |  | |  | |  | |  | |  | |  | |  | |  | |  | |  |  |
| Deletion | L729del |  | | | 729 | | VUS | |  | |  | |  | |  | |  | |  | |  | |  | |  | |  | |  | |  | |  | |  | |  |  |
| Frameshift | M734IfsX11 | c.2201dupT | | | 734 | | LP | |  | |  | |  | |  | | - | |  | |  | |  | |  | |  | |  | |  | |  | |  | |  |  |
| Missense | M734V |  | | | 734 | | VUS | |  | |  | |  | |  | | - | |  | |  | |  | |  | |  | | 0.7 | |  | | No effect | | 1 | | -8 | Slower |
| Missense | A735V |  | | | 735 | | VUS | | + | | - | | + | | + | | - | | - | | - | | - | | - | |  | | 0.25 | |  | | No effect | | 23 | | -7 |  |
| Missense | A735E |  | | | 735 | | VUS | | + | | - | | + | | - | | - | | - | | - | | - | | - | |  | | 0 | |  | |  | |  | |  |  |
| Missense | A735T |  | | | 735 | | LP | | - | | - | | - | | + | | - | | - | | - | | - | | - | |  | | 0.65 | |  | | No effect | | 5 | | 4 | Faster |
| Premature truncation | N740X |  | | | 740 | | LP | |  | |  | |  | |  | | - | |  | |  | |  | |  | |  | |  | |  | |  | |  | |  |  |
| Deletion | N740del |  | | | 740 | | VUS | |  | |  | |  | |  | |  | |  | |  | |  | |  | |  | |  | |  | |  | |  | |  |  |
| Missense | E746K |  | | | 746 | | VUS | |  | |  | |  | |  | | - | |  | |  | |  | |  | |  | | 0.4 | |  | | No effect | | 1 | | -2 | No effect |
| Missense | G752R |  | | | 752 | | P | | - | | + | | + | | + | | + | | - | | + | | - | | - | |  | | 0.05 | |  | |  | | 30 | | 10 |  |
| Duplication | exon15_28dup |  | | | 756 | | VUS | |  | |  | |  | |  | |  | |  | |  | |  | |  | |  | |  | |  | |  | |  | |  |  |
| Frameshift | F756LfsX8 |  | | | 756 | | LP | |  | |  | |  | |  | |  | |  | |  | |  | |  | |  | |  | |  | |  | |  | |  |  |
| Missense | G758E |  | | | 758 | | VUS | |  | |  | |  | |  | | - | |  | |  | |  | |  | |  | |  | |  | |  | |  | |  |  |
| Frameshift | I759FfsX6 | c.2274delG | | | 759 | | LP | |  | |  | |  | |  | | - | |  | |  | |  | |  | |  | |  | |  | |  | |  | |  |  |
| Missense | M764R |  | | | 764 | | VUS | |  | |  | |  | |  | | - | |  | |  | |  | |  | |  | |  | |  | |  | |  | |  |  |
| Missense | M764K |  | | | 764 | | VUS | | + | | + | | + | | + | | + | | + | | - | | - | | - | |  | | 0.8 | |  | | Slower | | 4 | | 3 | No effect |
| Missense | R769C |  | | | 769 | | VUS | |  | |  | |  | | + | | + | |  | |  | |  | |  | |  | |  | |  | |  | |  | |  |  |
| Missense | D772N |  | | | 772 | | VUS | |  | |  | |  | | - | | - | |  | |  | |  | |  | |  | | 1.05 | |  | | No effect | | -6 | | -1 | No effect |
| Missense | P773S |  | | | 773 | | VUS | |  | |  | |  | |  | | - | |  | |  | |  | |  | |  | | 1.2 | |  | | No effect | | 2 | | -4 | Slower |
| Frameshift | Y774TfsX28 | c.2320delT | | | 774 | | LP | |  | |  | |  | | - | | - | |  | |  | |  | |  | |  | |  | |  | |  | |  | |  |  |
| Deletion | Y776del |  | | | 776 | | VUS | |  | |  | |  | |  | | - | |  | |  | |  | |  | |  | |  | |  | |  | |  | |  |  |
| Missense | F777L |  | | | 777 | | VUS | |  | |  | |  | |  | |  | |  | |  | |  | |  | |  | |  | |  | |  | |  | |  |  |
| Missense | Q779K |  | | | 779 | | VUS | | - | |  | |  | |  | |  | |  | |  | |  | |  | |  | |  | |  | |  | |  | |  |  |
| Premature truncation | Q779X |  | | | 779 | | LP | |  | |  | |  | |  | | + | |  | |  | |  | |  | |  | |  | |  | |  | |  | |  |  |
| Premature truncation | W781X |  | | | 781 | | P | | + | | - | |  | | + | | + | |  | |  | |  | |  | |  | |  | |  | |  | |  | |  |  |
| Missense | N782T |  | | | 782 | | VUS | |  | |  | |  | |  | | - | |  | |  | |  | |  | |  | |  | |  | |  | |  | |  |  |
| Missense | D785N |  | | | 785 | | LP | | - | | - | | - | | + | | - | | - | | - | | - | | - | |  | | 0.4 | |  | | Slower | | 12 | | -7 | Slower |
| Missense | V789I |  | | | 789 | | VUS | |  | |  | |  | |  | | - | |  | |  | |  | |  | |  | |  | |  | |  | |  | |  |  |
| Frameshift | S792KfsX158 |  | | | 792 | | LP | |  | |  | |  | |  | | - | |  | |  | |  | |  | |  | |  | |  | |  | |  | |  |  |
| Missense | R808C |  | | | 808 | | VUS | | - | | - | | - | | + | | - | | - | | - | | - | | - | |  | | 0.2 | |  | | No effect | | 1 | | -9 | Slower |
| Missense | R808P |  | | | 808 | | VUS | |  | |  | |  | |  | | - | |  | |  | |  | |  | |  | |  | |  | |  | |  | |  |  |
| Missense | R811H |  | | | 811 | | VUS | | + | | + | | + | | + | | + | | - | | + | | - | | + | |  | | 0.4 | |  | | Slower | | 0 | | -8 | Slower |
| Missense | L812Q |  | | | 812 | | P | | - | | - | | - | | + | | - | | - | | - | | - | | - | |  | | 0.45 | | 0.6 | | No effect | | 0 | | -20 | No effect |
| Missense | L812P | c.2435_2436+3delTGGTAinsCGCCT | | | 812 | | LP | |  | |  | |  | |  | | - | |  | |  | |  | |  | |  | |  | |  | |  | |  | |  |  |
| Missense | R814Q |  | | | 814 | | VUS | | + | | - | | + | | + | | - | | - | | - | | - | | - | | + | | 1.15 | |  | | No effect | | -2 | | 0 | No effect |
| Missense | K817E |  | | | 817 | | LP | | - | | + | | + | | + | | + | | + | | - | | - | | - | |  | | 0.5 | |  | | Slower | | 24 | | 0 | Slower |
| Missense | W822C |  | | | 822 | | VUS | | + | | - | |  | |  | | - | |  | |  | |  | |  | |  | |  | |  | |  | |  | |  |  |
| Premature truncation | W822X |  | | | 822 | | P | | + | | + | | + | | + | | - | | - | | - | | - | | - | |  | | 0 | | 0.55 | |  | | 0 | | 0 |  |
| Missense | S835L |  | | | 835 | | VUS | | - | | - | |  | | - | | - | |  | |  | |  | |  | |  | |  | |  | |  | |  | |  |  |
| Missense | L839P | c.2533delG | | | 839 | | LP | | + | | + | | + | | + | | + | | - | | + | | - | | + | |  | | 0.05 | |  | |  | |  | |  |  |
| Frameshift | V845CfsX2 |  | | | 845 | | LP | |  | |  | |  | |  | | - | |  | |  | |  | |  | |  | |  | |  | |  | |  | |  |  |
| Missense | L846R |  | | | 846 | | VUS | | + | |  | |  | |  | |  | |  | |  | |  | |  | |  | |  | |  | |  | |  | |  |  |
| Frameshift | I848SfsX33 | c.2550_2551dupGT | | | 848 | | P | | + | | - | | + | | + | | - | | - | | - | | - | | - | |  | | 0.05 | |  | |  | |  | |  |  |
| Frameshift | F851CfsX19 | c.2549_2550insTG | | | 851 | | LP | |  | |  | |  | |  | | - | |  | |  | |  | |  | |  | |  | |  | |  | |  | |  |  |
| Frameshift | F851GfsX19 |  | | | 851 | | LP | |  | |  | |  | |  | | - | |  | |  | |  | |  | |  | |  | |  | |  | |  | |  |  |
| Missense | F851L |  | | | 851 | | VUS | |  | |  | |  | |  | | - | |  | |  | |  | |  | |  | | 0.15 | |  | | No effect | | 4 | | 0 | No effect |
| Frameshift | F853LfsX16 | c.2559delT | | | 853 | | LP | |  | |  | |  | |  | | - | |  | |  | |  | |  | |  | |  | |  | |  | |  | |  |  |
| Frameshift | L860fsx89 |  | | | 860 | | LP | |  | |  | |  | | - | | - | |  | |  | |  | |  | |  | |  | |  | |  | |  | |  |  |
| Frameshift | F861fs951X | c.2582_2583delTT | | | 861 | | P | | - | | + | | + | | - | | + | | + | | + | | - | | - | |  | |  | |  | |  | |  | |  |  |
| Frameshift | F861WfsX90 |  | | | 861 | | P | | - | | - | | - | | + | | - | | - | | - | | - | | - | |  | |  | |  | |  | |  | |  |  |
| Missense | E867Q |  | | | 867 | | VUS | |  | |  | |  | |  | | - | |  | |  | |  | |  | |  | |  | |  | |  | |  | |  |  |
| Premature truncation | E867X |  | | | 867 | | P | | - | | + | | - | |  | | + | | - | | + | | - | | - | |  | |  | |  | |  | |  | |  |  |
| Premature truncation | L867X | c.2602delC | | | 867 | | LP | |  | |  | |  | |  | |  | |  | |  | |  | |  | |  | |  | |  | |  | |  | |  |  |
| Premature truncation | L868X |  | | | 868 | | LP | |  | |  | |  | |  | | - | |  | |  | |  | |  | |  | |  | |  | |  | |  | |  |  |
| Missense | D870H |  | | | 870 | | VUS | | + | | - | |  | |  | | - | |  | |  | |  | |  | |  | |  | |  | |  | |  | |  |  |
| Frameshift | S871fs+9X |  | | | 871 | | LP | |  | |  | |  | |  | | - | |  | |  | |  | |  | |  | |  | |  | |  | |  | |  |  |
| Missense | R878C |  | | | 878 | | LP | | + | | + | | + | | + | | + | | + | | - | | - | | + | |  | | 0 | | 0.5 | | No effect | | 0 | | 0 | No effect |
| Missense | R878H |  | | | 878 | | LP | |  | |  | |  | |  | | - | |  | |  | |  | |  | |  | | 0 | |  | |  | |  | |  |  |
| Missense | W879R |  | | | 879 | | VUS | |  | |  | |  | |  | |  | |  | |  | |  | |  | |  | | 0 | |  | |  | |  | |  |  |
| Missense | H886P |  | | | 886 | | VUS | |  | |  | |  | |  | | - | |  | |  | |  | |  | |  | |  | |  | |  | |  | |  |  |
| Missense | H886Q |  | | | 886 | | VUS | | - | | + | | + | |  | | + | | - | | + | | - | | + | |  | |  | |  | |  | |  | |  |  |
| Missense | I890T |  | | | 890 | | P | | - | | - | | - | | + | | - | | - | | - | | - | | - | |  | | 0.7 | |  | |  | | 5 | | 0 | No effect |
| Missense | F891I |  | | | 891 | | VUS | |  | |  | |  | |  | | - | |  | |  | |  | |  | |  | |  | |  | |  | |  | |  |  |
| Missense | F892I |  | | | 892 | | VUS | | - | | - | |  | | - | | - | |  | |  | |  | |  | |  | | 0 | |  | |  | |  | |  |  |
| Missense | R893C |  | | | 893 | | VUS | | - | | + | | + | |  | | + | | + | | - | | - | | - | |  | |  | |  | |  | |  | |  |  |
| Missense | R893H |  | | | 893 | | VUS | | + | | + | | + | | + | | - | | - | | - | | - | | - | |  | |  | |  | |  | |  | |  |  |
| Missense | C896S |  | | | 896 | | VUS | |  | |  | |  | |  | | - | |  | |  | |  | |  | |  | |  | |  | |  | |  | |  |  |
| Missense | E901K |  | | | 901 | | LP | | - | | - | | - | | + | | - | | - | | - | | - | | - | |  | | 0.05 | |  | | No effect | | 4 | |  |  |
| Premature truncation | W904X |  | | | 904 | | LP | | - | |  | |  | |  | |  | |  | |  | |  | |  | |  | |  | |  | |  | |  | |  |  |
| Missense | S910L |  | | | 910 | | VUS | | + | | + | | + | | - | | + | | + | | - | | - | | + | |  | | 0 | | 0.5 | |  | |  | |  |  |
| Missense | C915R |  | | | 915 | | VUS | |  | |  | |  | |  | | - | |  | |  | |  | |  | |  | |  | |  | |  | |  | |  |  |
| Missense | L917R |  | | | 917 | | VUS | |  | |  | |  | |  | | - | |  | |  | |  | |  | |  | |  | |  | |  | |  | |  |  |
| Missense | N927S |  | | | 927 | | VUS | |  | |  | |  | | - | | - | |  | |  | |  | |  | |  | | 0.3 | |  | |  | |  | |  |  |
| Missense | L928P |  | | | 928 | | VUS | |  | |  | |  | |  | | - | |  | |  | |  | |  | |  | | 0 | |  | |  | |  | |  |  |
| Missense | L935P |  | | | 935 | | VUS | |  | |  | |  | |  | | - | |  | |  | |  | |  | |  | |  | |  | |  | |  | |  |  |
| Premature truncation | P950X |  | | | 950 | | LP | |  | |  | |  | |  | | - | |  | |  | |  | |  | |  | |  | |  | |  | |  | |  |  |
| Frameshift | D951MfsX6 | c.2850delT | | | 951 | | LP | |  | |  | |  | |  | | - | |  | |  | |  | |  | |  | |  | |  | |  | |  | |  |  |
| Premature truncation | D951X |  | | | 951 | | LP | |  | |  | |  | |  | | - | |  | |  | |  | |  | |  | |  | |  | |  | |  | |  |  |
| Missense | R965H |  | | | 965 | | VUS | |  | |  | |  | | - | | - | |  | |  | |  | |  | |  | |  | | 0.8 | |  | |  | | 0 |  |
| Missense | R965C |  | | | 965 | | VUS | | + | | - | | + | | + | | - | | - | | - | | - | | - | |  | | 1 | |  | | No effect | | 0 | | -9 | Slower |
| Frameshift | F972GfsX170 | c.2914_2923delTTTGTCAAGC | | | 972 | | LP | |  | |  | |  | |  | | - | |  | |  | |  | |  | |  | |  | |  | |  | |  | |  |  |
| Missense | K974D |  | | | 974 | | VUS | | - | | - | |  | | + | | - | |  | |  | |  | |  | |  | |  | |  | |  | |  | |  |  |
| Missense | R988W |  | | | 988 | | VUS | |  | |  | |  | |  | | - | |  | |  | |  | |  | |  | |  | |  | |  | |  | |  |  |
| Missense | R988Q |  | | | 988 | | VUS | | - | |  | |  | |  | |  | |  | |  | |  | |  | |  | |  | |  | |  | |  | |  |  |
| Missense | A997T |  | | | 997 | | LB | |  | |  | |  | |  | | - | |  | |  | |  | |  | |  | |  | |  | |  | |  | |  |  |
| Missense | Q1000K |  | | | 1000 | | VUS | | + | | - | | + | | + | | - | | - | | - | | - | | - | |  | |  | |  | |  | |  | |  |  |
| Frameshift | P1002HfsX25 | c.3005-3012delCCAGCTGG | | | 1002 | | LP | | - | | - | | - | | + | | - | | - | | - | | - | | - | |  | |  | |  | |  | |  | |  |  |
| Missense | P1014S |  | | | 1014 | | VUS | |  | |  | |  | |  | |  | |  | |  | |  | |  | |  | | 1.2 | |  | | No effect | | -10 | | 0 | No effect |
| Frameshift | c.3045_3046delGA |  | | | 1016 | | VUS | |  | |  | |  | |  | |  | |  | |  | |  | |  | |  | |  | |  | |  | |  | |  |  |
| Missense | R1023H |  | | | 1023 | | LB | | + | | - | | + | | + | | - | | - | | - | | - | | - | |  | | 0.85 | | 0.6 | | Slower | | 0 | | 0 | No effect |
| Missense | D1041N |  | | | 1041 | | VUS | |  | |  | |  | |  | | - | |  | |  | |  | |  | |  | |  | |  | |  | |  | |  |  |
| Frameshift | P1048insTG/fsX98 | c.3140_3141dupTG and c.3142_3143insTG | | | 1048 | | LP | |  | |  | |  | | - | | - | |  | |  | |  | |  | |  | |  | |  | |  | |  | |  |  |
| Missense | E1053K |  | | | 1053 | | VUS | | + | | + | | + | | + | | - | | - | | - | | - | | - | | + | | 1 | | 0.7 | | Faster | | 8 | | -4 | Slower |
| Missense | D1055G |  | | | 1055 | | VUS | |  | |  | |  | |  | | - | |  | |  | |  | |  | |  | |  | |  | |  | |  | |  |  |
| Frameshift | D1057EfsX88 | c.3171_3172delTGinsA | | | 1057 | | LP | |  | |  | |  | |  | | - | |  | |  | |  | |  | |  | |  | |  | |  | |  | |  |  |
| Premature truncation | Q1059X |  | | | 1059 | | LP | | + | | - | |  | |  | | - | |  | |  | |  | |  | |  | |  | |  | |  | |  | |  |  |
| Non-coding / Splice | c.3228+2delT |  | | | 1077 | | VUS | |  | |  | |  | | - | | - | |  | |  | |  | |  | |  | |  | |  | |  | |  | |  |  |
| Missense | S1079Y |  | | | 1079 | | VUS | |  | |  | |  | |  | | - | |  | |  | |  | |  | |  | |  | |  | |  | |  | |  |  |
| Frameshift | E1087PfsX57 |  | | | 1087 | | LP | |  | |  | |  | |  | | - | |  | |  | |  | |  | |  | |  | |  | |  | |  | |  |  |
| Missense | P1090L |  | | | 1090 | | VUS | | + | | + | | + | | + | | + | | - | | + | | - | | - | |  | | 1 | |  | |  | | 0 | | 0 |  |
| Premature truncation | W1095X |  | | | 1095 | | LP | | - | | - | | - | | - | | - | | - | | - | | - | | - | |  | |  | |  | |  | |  | |  |  |
| Non-coding / Splice | c.3288+2delT |  | | | 1097 | | VUS | |  | |  | |  | |  | |  | |  | |  | |  | |  | |  | |  | |  | |  | |  | |  |  |
| Missense | A1113V |  | | | 1113 | | B | |  | |  | |  | |  | | - | |  | |  | |  | |  | |  | | 0 | | 1 | |  | |  | | 0 |  |
| Missense | D1114N |  | | | 1114 | | VUS | |  | |  | |  | | - | | - | |  | |  | |  | |  | | + | |  | |  | |  | |  | |  |  |
| Premature truncation | W1115X |  | | | 1115 | | LP | |  | |  | |  | |  | | - | |  | |  | |  | |  | |  | |  | |  | |  | |  | |  |  |
| Premature truncation | Q1118X |  | | | 1118 | | LP | | - | | - | |  | |  | | - | |  | |  | |  | |  | |  | |  | |  | |  | |  | |  |  |
| Frameshift | A1123GfsX16 |  | | | 1123 | | P | | - | | - | |  | |  | | + | |  | |  | |  | |  | |  | |  | |  | |  | |  | |  |  |
| Non-coding / Splice | c.3390-1G>A |  | | | 1131 | | VUS | |  | |  | |  | |  | |  | |  | |  | |  | |  | |  | |  | |  | |  | |  | |  |  |
| Non-coding / Splice | c.3391-1G>A |  | | | 1131 | | VUS | |  | |  | |  | |  | |  | |  | |  | |  | |  | |  | |  | |  | |  | |  | |  |  |
| Missense | S1140T |  | | | 1140 | | LP | |  | |  | |  | |  | | - | |  | |  | |  | |  | |  | | 0 | | 0.5 | |  | |  | | 0 |  |
| Premature truncation | E1152X |  | | | 1152 | | P | | - | | - | |  | | + | | - | |  | |  | |  | |  | |  | |  | |  | |  | |  | |  |  |
| Frameshift | c.3480delT |  | | | 1161 | | LP | | + | | + | | + | | + | | - | | - | | - | | - | | - | |  | | 0 | | 0.5 | |  | |  | |  |  |
| Frameshift | Q1185GfsX55 | c.3553_3554delCA | | | 1185 | | LP | |  | |  | |  | |  | | - | |  | |  | |  | |  | |  | |  | |  | |  | |  | |  |  |
| Premature truncation | W1191X |  | | | 1191 | | P | | + | | + | | + | | + | | + | | + | | + | | - | | + | | + | | 0 | | 0.5 | |  | | 0 | | 0 |  |
| Missense | R1192Q |  | | | 1192 | | VUS | | + | | + | | + | | - | | - | | - | | - | | - | | - | |  | | 1 | |  | | Faster | | 0 | | 4 | No effect |
| Premature truncation | W1192X |  | | | 1192 | | LP | |  | |  | |  | |  | |  | |  | |  | |  | |  | |  | |  | |  | |  | |  | |  |  |
| Missense | R1193Q |  | | | 1193 | | VUS | | - | | + | | + | |  | | + | | - | | + | | + | | - | | + | | 1 | |  | | No effect | | 2 | | -5 |  |
| Missense | R1195H |  | | | 1195 | | VUS | | - | |  | |  | |  | |  | |  | |  | |  | |  | |  | |  | |  | |  | |  | |  |  |
| Missense | H1200Y |  | | | 1200 | | VUS | | - | |  | |  | |  | |  | |  | |  | |  | |  | |  | |  | |  | |  | |  | |  |  |
| Premature truncation | E1208X |  | | | 1208 | | LP | | + | | + | |  | | + | | - | |  | |  | |  | |  | |  | |  | |  | |  | |  | |  |  |
| Deletion | I1212del |  | | | 1212 | | VUS | |  | |  | |  | |  | | - | |  | |  | |  | |  | |  | |  | |  | |  | |  | |  |  |
| Missense | S1218I |  | | | 1218 | | P | | - | | + | | + | | + | | + | | - | | + | | - | | + | |  | | 0 | | 0.5 | |  | |  | |  |  |
| Missense | S1219N |  | | | 1219 | | VUS | |  | |  | |  | |  | | - | |  | |  | |  | |  | |  | |  | |  | |  | |  | |  |  |
| Frameshift | A1223PfsX7 | c.3667delG | | | 1223 | | LP | |  | |  | |  | |  | | - | |  | |  | |  | |  | |  | |  | |  | |  | |  | |  |  |
| Missense | E1225K |  | | | 1225 | | VUS | | + | | - | | + | | - | | + | | + | | + | | - | | - | |  | | 0.35 | |  | | No effect | | 4 | | 12 | Faster |
| Missense | Y1228H |  | | | 1228 | | VUS | | - | | - | |  | | + | | - | |  | |  | |  | |  | |  | |  | |  | |  | |  | |  |  |
| Missense | R1232W |  | | | 1232 | | VUS | | - | | - | | - | | + | | - | | - | | - | | - | | - | |  | | 1 | |  | | No effect | | 0 | | -6 | Faster |
| Missense | R1232Q |  | | | 1232 | | VUS | |  | |  | |  | |  | | - | |  | |  | |  | |  | |  | |  | |  | |  | |  | |  |  |
| Missense | K1236N |  | | | 1236 | | VUS | |  | |  | |  | |  | | - | |  | |  | |  | |  | |  | |  | |  | |  | |  | |  |  |
| Missense | K1236R |  | | | 1236 | | VUS | |  | |  | |  | |  | |  | |  | |  | |  | |  | |  | |  | |  | |  | |  | |  |  |
| Missense | L1239P |  | | | 1239 | | VUS | |  | |  | |  | |  | | - | |  | |  | |  | |  | |  | | 0 | | 0.3 | |  | | 10 | | 0 |  |
| Missense | E1240Q |  | | | 1240 | | VUS | |  | |  | |  | |  | | - | |  | |  | |  | |  | |  | |  | |  | |  | |  | |  |  |
| Missense | D1243N |  | | | 1243 | | VUS | | - | | - | |  | | - | | - | |  | |  | |  | |  | |  | | 1.15 | |  | | Slower | | 6 | | 8 | Faster |
| Missense | T1247I |  | | | 1247 | | VUS | | + | |  | |  | |  | |  | |  | |  | |  | |  | |  | |  | |  | |  | |  | |  |  |
| Missense | V1249D |  | | | 1249 | | VUS | |  | |  | |  | |  | | - | |  | |  | |  | |  | |  | |  | |  | |  | |  | |  |  |
| Missense | E1253G |  | | | 1253 | | VUS | |  | |  | |  | |  | | - | |  | |  | |  | |  | |  | |  | |  | |  | |  | |  |  |
| Missense | G1262S |  | | | 1262 | | VUS | | - | | + | | + | | + | | + | | + | | + | | - | | + | | + | | 0.45 | |  | | No effect | | -12 | | -2 | Slower |
| Missense | N1269S |  | | | 1269 | | VUS | | - | | - | |  | |  | | - | |  | |  | |  | |  | |  | |  | |  | |  | |  | |  |  |
| Missense | W1271C |  | | | 1271 | | VUS | |  | |  | |  | |  | | - | |  | |  | |  | |  | |  | |  | |  | |  | |  | |  |  |
| Premature truncation | c.3816delG |  | | | 1273 | | P | | + | | - | |  | | + | | - | |  | |  | |  | |  | |  | |  | |  | |  | |  | |  |  |
| Missense | D1275N |  | | | 1275 | | VUS | | + | | - | | + | |  | | + | | + | | + | | + | | - | |  | | 0.75 | |  | | No effect | | 10 | | 0 | No effect |
| Missense | V1281F |  | | | 1281 | | VUS | | - | | - | | - | | + | | - | | - | | - | | - | | - | |  | | 1 | |  | | No effect | | -3 | | -1 | No effect |
| Deletion | exon23_deletion |  | | | 1281 | | VUS | |  | |  | |  | |  | |  | |  | |  | |  | |  | |  | |  | |  | |  | |  | |  |  |
| Non-coding / Splice | c.3840+1G>A |  | | | 1281 | | VUS | | + | | + | | + | | + | | + | | + | | - | | - | | + | |  | |  | |  | |  | |  | |  |  |
| Missense | A1288G |  | | | 1288 | | VUS | |  | |  | |  | |  | | - | |  | |  | |  | |  | |  | |  | |  | |  | |  | |  |  |
| Missense | F1293S |  | | | 1293 | | VUS | |  | |  | |  | |  | |  | |  | |  | |  | |  | |  | | 0.45 | |  | |  | | 7 | | 0 |  |
| Missense | A1294G |  | | | 1294 | | VUS | | - | | - | | - | | + | | + | | - | | + | | + | | - | |  | | 0.8 | |  | |  | | -2 | | -6 | No effect |
| Frameshift | M1296fs |  | | | 1299 | | LP | |  | |  | |  | |  | | - | |  | |  | |  | |  | |  | |  | |  | |  | |  | |  |  |
| Frameshift | I1299SfsX13 | c.3894delC | | | 1299 | | LP | |  | |  | |  | |  | | - | |  | |  | |  | |  | |  | |  | |  | |  | |  | |  |  |
| Missense | T1304M |  | | | 1304 | | VUS | | + | | - | |  | | - | | + | |  | |  | |  | |  | |  | |  | |  | |  | |  | |  |  |
| Missense | L1311P |  | | | 1311 | | VUS | |  | |  | |  | |  | | - | |  | |  | |  | |  | |  | |  | |  | |  | |  | |  |  |
| Frameshift | c.3940_3941delCT |  | | | 1314 | | VUS | |  | |  | |  | |  | |  | |  | |  | |  | |  | |  | |  | |  | |  | |  | |  |  |
| Premature truncation | R1316X |  | | | 1316 | | LP | | - | | - | | - | | - | | - | | - | | - | | - | | - | |  | |  | |  | |  | |  | |  |  |
| Missense | G1319V |  | | | 1319 | | VUS | | + | | - | | + | | + | | + | | - | | + | | - | | + | |  | | 1 | | 1 | | No effect | | 4 | | -6 | Slower |
| Non-coding / Splice | c.3963+2T>C |  | | | 1322 | | VUS | | - | | - | |  | | + | | + | |  | |  | |  | |  | |  | |  | |  | |  | |  | |  |  |
| Non-coding / Splice | c.3963+4A>G |  | | | 1322 | | VUS | |  | |  | |  | |  | | - | |  | |  | |  | |  | |  | |  | |  | |  | |  | |  |  |
| Missense | V1323G |  | | | 1323 | | VUS | |  | |  | |  | |  | | - | |  | |  | |  | |  | |  | |  | |  | |  | |  | |  |  |
| Missense | V1328M |  | | | 1328 | | VUS | | - | | - | | - | | - | | - | | - | | - | | - | | - | |  | | 1 | |  | |  | | 0 | | 7 | No effect |
| Missense | P1332L |  | | | 1332 | | VUS | |  | |  | |  | |  | | - | |  | |  | |  | |  | |  | |  | |  | |  | |  | |  |  |
| Missense | M1335R |  | | | 1335 | | VUS | |  | |  | |  | |  | | - | |  | |  | |  | |  | |  | |  | |  | |  | |  | |  |  |
| Missense | V1340I |  | | | 1340 | | VUS | | + | | + | | + | | + | | - | | - | | - | | - | | - | |  | | 0 | |  | |  | | 0 | | 0 | Faster |
| Missense | F1344S |  | | | 1344 | | VUS | | + | | + | | + | | + | | - | | - | | - | | - | | - | |  | | 1 | |  | |  | | 10 | | 0 | Faster |
| Missense | F1344L |  | | | 1344 | | VUS | |  | |  | |  | |  | | - | |  | |  | |  | |  | |  | |  | |  | |  | |  | |  |  |
| Missense | W1345C |  | | | 1345 | | VUS | | - | | - | | - | | + | | - | | - | | - | | - | | - | |  | | 0.1 | |  | |  | |  | |  |  |
| Missense | L1346I |  | | | 1346 | | VUS | |  | |  | |  | |  | | - | |  | |  | |  | |  | |  | |  | |  | |  | |  | |  |  |
| Missense | L1346P |  | | | 1346 | | VUS | |  | |  | |  | | - | | - | |  | |  | |  | |  | |  | | 0 | |  | |  | |  | |  |  |
| Missense | I1350T |  | | | 1350 | | VUS | |  | |  | |  | |  | | + | |  | |  | |  | |  | |  | |  | |  | |  | |  | |  |  |
| Missense | M1351R |  | | | 1351 | | VUS | |  | |  | |  | |  | | - | |  | |  | |  | |  | |  | |  | |  | |  | |  | |  |  |
| Missense | V1353M |  | | | 1353 | | VUS | |  | |  | |  | |  | | - | |  | |  | |  | |  | |  | | 1.05 | |  | | No effect | | 4 | | 2 | No effect |
| Premature truncation | c.4066_4088delTT |  | | | 1356 | | P | | - | | - | |  | | + | | - | |  | |  | |  | |  | |  | |  | |  | |  | |  | |  |  |
| Missense | G1358W |  | | | 1358 | | VUS | |  | |  | |  | |  | | - | |  | |  | |  | |  | |  | |  | |  | |  | |  | |  |  |
| Missense | K1359N |  | | | 1359 | | VUS | |  | |  | |  | |  | | - | |  | |  | |  | |  | |  | |  | |  | |  | |  | |  |  |
| Missense | F1360C |  | | | 1360 | | VUS | |  | |  | |  | |  | | - | |  | |  | |  | |  | |  | |  | |  | |  | |  | |  |  |
| Frameshift | R1361SfsX12 | c.4083delG | | | 1362 | | LP | |  | |  | |  | |  | |  | |  | |  | |  | |  | |  | |  | |  | |  | |  | |  |  |
| Missense | C1363Y |  | | | 1363 | | VUS | |  | |  | |  | | - | | - | |  | |  | |  | |  | |  | |  | |  | |  | |  | |  |  |
| Missense | N1365S |  | | | 1365 | | VUS | |  | |  | |  | |  | |  | |  | |  | |  | |  | |  | |  | |  | |  | |  | |  |  |
| Premature truncation | L1373X | c.4118delT | | | 1373 | | LP | |  | |  | |  | | - | | - | |  | |  | |  | |  | |  | |  | |  | |  | |  | |  |  |
| Missense | V1378M |  | | | 1378 | | LP | | - | | + | | + | | + | | - | | - | | - | | - | | - | |  | | 0.3 | |  | |  | | 0 | | 0 |  |
| Missense | N1380K |  | | | 1380 | | VUS | | - | | + | | + | | + | | + | | - | | + | | - | | - | |  | | 0 | |  | |  | |  | |  |  |
| Deletion | N1380del |  | | | 1380 | | VUS | |  | |  | |  | |  | |  | |  | |  | |  | |  | |  | |  | |  | |  | |  | |  |  |
| Missense | S1382I |  | | | 1382 | | VUS | | - | | - | |  | | + | | + | |  | |  | |  | |  | |  | | 0.05 | |  | | Slower | | -4 | |  | Faster |
| Premature truncation | Q1383X |  | | | 1383 | | LP | |  | |  | |  | |  | | - | |  | |  | |  | |  | |  | |  | |  | |  | |  | |  |  |
| Premature truncation | L1393X |  | | | 1393 | | P | | - | | - | | - | | + | | + | | - | | - | | - | | + | |  | | 0 | | 0.55 | |  | | 0 | | 0 |  |
| Premature truncation | Y1394X |  | | | 1394 | | LP | |  | |  | |  | |  | | - | |  | |  | |  | |  | |  | |  | |  | |  | |  | |  |  |
| Insertion | K1397R+1X | c.4190delA | | | 1397 | | LP | | - | | - | |  | |  | | - | |  | |  | |  | |  | |  | |  | |  | |  | |  | |  |  |
| Frameshift | c.4189delT |  | | | 1397 | | VUS | |  | |  | |  | |  | |  | |  | |  | |  | |  | |  | |  | |  | |  | |  | |  |  |
| Premature truncation | V1398X |  | | | 1398 | | LP | |  | |  | |  | |  | | - | |  | |  | |  | |  | |  | |  | |  | |  | |  | |  |  |
| Missense | V1405L |  | | | 1405 | | VUS | |  | |  | |  | | - | | - | |  | |  | |  | |  | |  | | 0.15 | |  | | No effect | | -3 | | -2 |  |
| Missense | V1405M |  | | | 1405 | | VUS | | - | | - | | - | |  | | - | | - | | - | | - | | - | |  | | 0.35 | |  | | No effect | | 10 | |  |  |
| Missense | G1406R |  | | | 1406 | | P | | - | | + | | + | | + | | + | | - | | + | | - | | + | |  | | 0.1 | |  | | No effect | | 0 | | -7 | Slower |
| Missense | G1406E |  | | | 1406 | | VUS | |  | |  | |  | |  | | - | |  | |  | |  | |  | |  | | 0.35 | |  | | No effect | | -6 | | -3 | No effect |
| Missense | G1408R |  | | | 1408 | | LP | | - | | + | |  | | + | | + | |  | |  | |  | |  | |  | |  | |  | |  | |  | |  |  |
| Missense | Y1409C |  | | | 1409 | | VUS | |  | |  | |  | |  | | - | |  | |  | |  | |  | |  | |  | |  | |  | |  | |  |  |
| Premature truncation | Y1409X |  | | | 1409 | | LP | | - | |  | |  | |  | | - | |  | |  | |  | |  | |  | |  | |  | |  | |  | |  |  |
| Missense | L1412F |  | | | 1412 | | VUS | |  | |  | |  | |  | | - | |  | |  | |  | |  | |  | |  | |  | |  | |  | |  |  |
| Missense | A1416E |  | | | 1416 | | VUS | |  | |  | |  | |  | | - | |  | |  | |  | |  | |  | |  | |  | |  | |  | |  |  |
| Non-coding / Splice | c.4245+1G>A |  | | | 1416 | | VUS | |  | |  | |  | |  | |  | |  | |  | |  | |  | |  | |  | |  | |  | |  | |  |  |
| Missense | K1419E |  | | | 1419 | | VUS | |  | |  | |  | |  | | - | |  | |  | |  | |  | |  | |  | |  | |  | |  | |  |  |
| Missense | G1420R |  | | | 1420 | | VUS | | - | | - | | - | |  | | - | | - | | - | | - | | - | |  | | 0.05 | |  | |  | |  | |  |  |
| Missense | G1420V |  | | | 1420 | | VUS | |  | |  | |  | |  | | - | |  | |  | |  | |  | |  | | 0 | |  | |  | |  | |  |  |
| Missense | G1420P |  | | | 1420 | | VUS | |  | |  | |  | |  | |  | |  | |  | |  | |  | |  | |  | |  | |  | |  | |  |  |
| Missense | A1427S |  | | | 1427 | | VUS | |  | |  | |  | |  | | - | |  | |  | |  | |  | |  | |  | |  | |  | |  | |  |  |
| Missense | A1428S |  | | | 1428 | | LP | | + | | - | | + | | + | | - | | - | | - | | - | | - | |  | | 0.6 | |  | |  | | 0 | | 0 | No effect |
| Missense | A1428V |  | | | 1428 | | VUS | | - | | + | | + | | + | | - | | - | | - | | - | | - | |  | | 0 | |  | |  | |  | |  |  |
| Missense | R1432G |  | | | 1432 | | LP | | + | | - | | + | | + | | + | | - | | + | | - | | - | |  | | 0 | | 0.2 | |  | |  | |  |  |
| Missense | R1432S |  | | | 1432 | | VUS | | - | |  | |  | |  | | - | |  | |  | |  | |  | | + | |  | |  | |  | |  | |  |  |
| Missense | G1433V |  | | | 1433 | | VUS | |  | |  | |  | |  | | - | |  | |  | |  | |  | |  | |  | |  | |  | |  | |  |  |
| Missense | G1433W |  | | | 1433 | | VUS | | - | | - | | - | | + | | + | | + | | - | | - | | - | |  | |  | |  | |  | |  | |  |  |
| Premature truncation | Y1434X |  | | | 1434 | | LP | |  | |  | |  | |  | | - | |  | |  | |  | |  | |  | |  | |  | |  | |  | |  |  |
| Non-coding / Splice | c.4299+1delG |  | | | 1434 | | VUS | | + | | - | |  | |  | | - | |  | |  | |  | |  | |  | |  | |  | |  | |  | |  |  |
| Non-coding / Splice | c.4299+1G>T |  | | | 1434 | | VUS | |  | |  | |  | |  | | - | |  | |  | |  | |  | |  | |  | |  | |  | |  | |  |  |
| Non-coding / Splice | c.4300-1G>A |  | | | 1434 | | VUS | |  | |  | |  | |  | | - | |  | |  | |  | |  | |  | |  | |  | |  | |  | |  |  |
| Frameshift | c.4299_4300insG |  | | | 1434 | | VUS | |  | |  | |  | |  | |  | |  | |  | |  | |  | |  | |  | |  | |  | |  | |  |  |
| Non-coding / Splice | G1433G | c.4299G>A | | | 1434 | | VUS | |  | |  | |  | |  | |  | |  | |  | |  | |  | |  | |  | |  | |  | |  | |  |  |
| Non-coding / Splice | c.4300-1G>A |  | | | 1434 | | VUS | |  | |  | |  | |  | |  | |  | |  | |  | |  | |  | |  | |  | |  | |  | |  |  |
| Non-coding / Splice | c.4300-2A>T |  | | | 1434 | | VUS | |  | |  | |  | |  | |  | |  | |  | |  | |  | |  | |  | |  | |  | |  | |  |  |
| Missense | P1438L |  | | | 1438 | | VUS | | + | | + | | + | | + | | + | | - | | + | | - | | + | |  | | 0 | |  | |  | |  | |  |  |
| Premature truncation | W1440X |  | | | 1440 | | LP | | + | | - | |  | | + | | + | |  | |  | |  | |  | |  | |  | |  | |  | |  | |  |  |
| Missense | E1441Q |  | | | 1441 | | VUS | |  | |  | |  | |  | | - | |  | |  | |  | |  | |  | |  | |  | |  | |  | |  |  |
| Missense | N1443S |  | | | 1443 | | VUS | | - | | - | |  | | + | | - | |  | |  | |  | |  | |  | |  | |  | |  | |  | |  |  |
| Missense | I1448L |  | | | 1448 | | VUS | |  | |  | |  | |  | | - | |  | |  | |  | |  | |  | |  | |  | |  | |  | |  |  |
| Missense | I1448T |  | | | 1448 | | VUS | |  | |  | |  | |  | | - | |  | |  | |  | |  | |  | |  | |  | |  | |  | |  |  |
| Missense | Y1449C |  | | | 1449 | | LP | | - | | + | | + | | + | | + | | - | | + | | + | | + | |  | | 0.1 | |  | |  | |  | |  |  |
| Missense | Y1449S |  | | | 1449 | | VUS | |  | |  | |  | | - | | - | |  | |  | |  | |  | |  | |  | |  | |  | |  | |  |  |
| Missense | V1451D |  | | | 1451 | | VUS | |  | |  | |  | |  | | - | |  | |  | |  | |  | |  | |  | |  | |  | |  | |  |  |
| Frameshift | F1459SfsX3 | c.4376_4379delTCTT | | | 1459 | | LP | |  | |  | |  | |  | | - | |  | |  | |  | |  | |  | |  | |  | |  | |  | |  |  |
| Missense | T1461S |  | | | 1461 | | VUS | | - | | - | | - | | - | | + | | - | | + | | - | | - | |  | | 0.6 | |  | | No effect | | 1 | | 3 | Faster |
| Missense | N1463Y |  | | | 1463 | | VUS | |  | |  | |  | |  | | - | |  | |  | |  | |  | |  | |  | |  | |  | |  | |  |  |
| Frameshift | L1464WfsX5 | c.4389_4396delCCTCTTTA | | | 1464 | | LP | |  | |  | |  | |  | | - | |  | |  | |  | |  | |  | |  | |  | |  | |  | |  |  |
| Frameshift | c.4396_4397insG |  | | | 1466 | | VUS | |  | |  | |  | |  | |  | |  | |  | |  | |  | |  | |  | |  | |  | |  | |  |  |
| Frameshift | G1467fs+13X |  | | | 1467 | | LP | |  | |  | |  | |  | | - | |  | |  | |  | |  | |  | |  | |  | |  | |  | |  |  |
| Missense | V1468F |  | | | 1468 | | VUS | |  | |  | |  | |  | | - | |  | |  | |  | |  | |  | |  | |  | |  | |  | |  |  |
| Premature truncation | Q1476X |  | | | 1476 | | LP | |  | |  | |  | |  | |  | |  | |  | |  | |  | |  | |  | |  | |  | |  | |  |  |
| Deletion | K1479del |  | | | 1479 | | VUS | | - | | + | | + | | + | | + | | + | | - | | - | | - | |  | | 0.8 | |  | | Slower | | 12 | | 6 | Faster |
| Non-coding / Splice | c.4437+5G>A |  | | | 1480 | | VUS | | - | | - | |  | | - | | - | |  | |  | |  | |  | |  | |  | |  | |  | |  | |  |  |
| Non-coding / Splice | c.4438-1C>T |  | | | 1480 | | VUS | | - | | + | |  | | - | | - | |  | |  | |  | |  | |  | |  | |  | |  | |  | |  |  |
| Premature truncation | K1493X |  | | | 1493 | | LP | |  | |  | |  | |  | | - | |  | |  | |  | |  | |  | |  | |  | |  | |  | |  |  |
| Deletion | K1493del |  | | | 1493 | | VUS | | - | | - | | - | | + | | + | | + | | + | | - | | + | |  | | 0 | | 0.5 | |  | |  | |  |  |
| Deletion | K1500del |  | | | 1500 | | VUS | | + | | + | | + | | + | | + | | + | | + | | - | | + | | + | | 0.5 | |  | | Slower | | 3 | | -13 |  |
| Missense | L1501V |  | | | 1501 | | VUS | | - | | - | |  | | - | | - | |  | |  | |  | |  | | + | |  | | 0.5 | |  | | 0 | | 0 |  |
| Missense | G1502S |  | | | 1502 | | VUS | |  | |  | |  | |  | | - | |  | |  | |  | |  | |  | |  | | 0.8 | |  | |  | | 0 |  |
| Deletion | KPQ1505-1507del |  | | | 1505 | | VUS | |  | |  | |  | | + | | - | |  | |  | |  | |  | | + | |  | |  | |  | |  | |  |  |
| Missense | P1506S |  | | | 1506 | | VUS | | - | | + | | + | | + | | + | | - | | + | | - | | - | |  | | 0.5 | |  | | Faster | | 9 | | -14 | No effect |
| Missense | P1506T |  | | | 1506 | | VUS | |  | |  | |  | |  | | - | |  | |  | |  | |  | |  | |  | |  | |  | |  | |  |  |
| Missense | R1512W |  | | | 1512 | | VUS | | + | | + | | + | |  | | + | | + | | + | | - | | - | |  | | 0.7 | |  | | Slower | | 0 | | 0 | Slower |
| Missense | I1521K |  | | | 1521 | | VUS | |  | |  | |  | |  | | - | |  | |  | |  | |  | |  | |  | |  | |  | |  | |  |  |
| Missense | V1525M |  | | | 1525 | | VUS | |  | |  | |  | |  | | - | |  | |  | |  | |  | |  | |  | |  | |  | |  | |  |  |
| Missense | K1527R |  | | | 1527 | | VUS | |  | |  | |  | | + | | - | |  | |  | |  | |  | |  | |  | |  | |  | |  | |  |  |
| Missense | N1541D |  | | | 1541 | | VUS | | - | | - | | - | | + | | + | | + | | + | | - | | + | |  | | 0.75 | |  | |  | | 7 | | -26 | Slower |
| Missense | E1548K |  | | | 1548 | | VUS | |  | |  | |  | |  | | - | |  | |  | |  | |  | |  | |  | |  | |  | |  | |  |  |
| Missense | S1553R |  | | | 1553 | | VUS | | - | | - | |  | | - | | - | |  | |  | |  | |  | |  | |  | |  | |  | |  | |  |  |
| Frameshift | F1567CfsX221 |  | | | 1567 | | LP | | + | | - | | + | | + | | - | | - | | - | | - | | - | |  | |  | |  | |  | |  | |  |  |
| Missense | A1569P |  | | | 1569 | | VUS | |  | |  | |  | | + | | - | |  | |  | |  | |  | |  | |  | |  | |  | |  | |  |  |
| Insertion | 1570insI | c.4708_4710dupATC | | | 1570 | | LP | |  | | + | | + | | - | | + | | - | | - | | - | | - | |  | | 0 | |  | |  | |  | |  |  |
| Insertion | 1570insG |  | | | 1570 | | VUS | |  | |  | |  | |  | | - | |  | |  | |  | |  | |  | |  | |  | |  | |  | |  |  |
| Missense | F1571C |  | | | 1571 | | VUS | |  | |  | |  | |  | | - | |  | |  | |  | |  | |  | |  | |  | |  | |  | |  |  |
| Deletion | del1572-1604 |  | | | 1572 | | LP | | + | | + | | + | | + | | - | | - | | - | | - | | - | |  | | 0 | |  | |  | |  | |  |  |
| Non-coding / Splice | c.4719C>T |  | | | 1573 | | VUS | |  | |  | |  | | - | | - | |  | |  | |  | |  | |  | |  | |  | |  | |  | |  |  |
| Missense | E1574K |  | | | 1574 | | VUS | | - | | - | | - | | + | | - | | - | | - | | - | | - | |  | | 0.45 | |  | |  | | 10 | |  |  |
| Frameshift | K1578fs/52 |  | | | 1578 | | P | | - | | + | | + | | - | | + | | + | | - | | - | | - | |  | | 0 | | 0.5 | |  | | 0 | | 0 |  |
| Frameshift | L1579SfsX53 | c.4732_4733dupAA | | | 1579 | | VUS | |  | |  | |  | |  | |  | |  | |  | |  | |  | |  | |  | |  | |  | |  | |  |  |
| Missense | L1582P |  | | | 1582 | | VUS | |  | |  | |  | | - | | - | |  | |  | |  | |  | |  | |  | |  | |  | |  | |  |  |
| Missense | R1583C |  | | | 1583 | | VUS | |  | |  | |  | |  | | - | |  | |  | |  | |  | |  | | 0.8 | |  | | No effect | | -3 | | -1 | Faster |
| Missense | R1583H |  | | | 1583 | | VUS | |  | |  | |  | |  | | - | |  | |  | |  | |  | |  | |  | |  | |  | |  | |  |  |
| Premature truncation | W1591X |  | | | 1591 | | LP | |  | |  | |  | |  | | - | |  | |  | |  | |  | |  | |  | |  | |  | |  | |  |  |
| Missense | V1604M |  | | | 1604 | | VUS | |  | |  | |  | |  | | - | |  | |  | |  | |  | |  | |  | |  | |  | |  | |  |  |
| Non-coding / Splice | c.4810+3_4810+6dupGGGT | 1604 | VUS | - | | - | |  | | + | | + | |  | |  | |  | |  | |  | |  | |  | |  | |  | |  | |  | |  |  |  |
| Non-coding / Splice | c.4813+2_4813+5dupTGGG | 1605 | VUS |  | |  | |  | |  | | - | |  | |  | |  | |  | |  | |  | |  | |  | |  | |  | |  | |  |  |  |
| Non-coding / Splice | c.4813+3_4813+6dupGGGT | 1605 | VUS |  | |  | |  | |  | |  | |  | |  | |  | |  | |  | |  | |  | |  | |  | |  | |  | |  |  |  |
| Non-coding / Splice | c.4813+5insTGGG | exon27-5insTGGG | | | 1605 | | VUS | |  | |  | |  | |  | |  | |  | |  | |  | |  | |  | |  | |  | |  | |  | |  |  |
| Missense | Q1613L |  | | | 1613 | | VUS | |  | |  | |  | |  | | - | |  | |  | |  | |  | |  | |  | |  | |  | |  | |  |  |
| Premature truncation | Y1615X |  | | | 1615 | | LP | |  | |  | |  | |  | | - | |  | |  | |  | |  | |  | |  | |  | |  | |  | |  |  |
| Deletion | F1617del |  | | | 1617 | | VUS | | - | | - | |  | | + | | + | |  | |  | |  | |  | | + | |  | |  | |  | |  | |  |  |
| Frameshift | P1619RfsX12 | c.4856delC | | | 1619 | | LP | |  | |  | |  | |  | | - | |  | |  | |  | |  | |  | |  | |  | |  | |  | |  |  |
| Missense | T1620M |  | | | 1620 | | VUS | | - | | + | | + | | + | | - | | - | | - | | - | | - | |  | | 1 | |  | | No effect | | 0 | | -9 | Slower |
| Premature truncation | R1623X |  | | | 1623 | | P | | + | | + | | + | | + | | + | | + | | - | | - | | - | |  | | 0 | | 0.5 | |  | | 0 | | 0 |  |
| Frameshift | R1623Efs7 | c.4867delC | | | 1623 | | LP | | - | | - | |  | | + | | - | |  | |  | |  | |  | |  | |  | |  | |  | |  | |  |  |
| Missense | R1623Q |  | | | 1623 | | VUS | |  | |  | |  | |  | | - | |  | |  | |  | |  | |  | |  | |  | |  | |  | |  |  |
| Premature truncation | R1629X |  | | | 1629 | | P | | - | | - | | - | | + | | - | | - | | - | | - | | - | |  | | 0 | |  | |  | |  | |  |  |
| Missense | R1629Q |  | | | 1629 | | LP | | + | | - | | + | | + | | - | | - | | - | | - | | - | |  | | 1 | |  | | Faster | | 0 | | -20 | Slower |
| Missense | R1629G |  | | | 1629 | | VUS | |  | |  | |  | |  | | - | |  | |  | |  | |  | |  | |  | |  | |  | |  | |  |  |
| Missense | R1632C |  | | | 1632 | | LP | | - | | - | | - | | + | | + | | + | | - | | - | | + | |  | | 0.7 | |  | | No effect | | 0 | | -15 | Slower |
| Missense | R1632H |  | | | 1632 | | VUS | | - | | - | | - | | - | | + | | + | | - | | - | | - | |  | | 0.65 | |  | | Slower | | -11 | | -12 | Slower |
| Missense | R1632L |  | | | 1632 | | VUS | |  | |  | |  | |  | |  | |  | |  | |  | |  | |  | |  | |  | |  | |  | |  |  |
| Premature truncation | R1638X |  | | | 1638 | | LP | |  | |  | |  | | - | | - | |  | |  | |  | |  | |  | |  | |  | |  | |  | |  |  |
| Missense | G1642E |  | | | 1642 | | VUS | |  | |  | |  | |  | | - | |  | |  | |  | |  | |  | | 0.15 | |  | | No effect | | 4 | | 7 | Faster |
| Missense | R1644C |  | | | 1644 | | VUS | | + | | + | | + | | - | | - | | - | | - | | - | | - | |  | |  | |  | |  | |  | |  |  |
| Missense | R1644H |  | | | 1644 | | VUS | | - | |  | |  | |  | |  | |  | |  | |  | |  | |  | |  | |  | |  | |  | |  |  |
| Missense | A1649V |  | | | 1649 | | VUS | | - | | - | |  | | + | | - | |  | |  | |  | |  | |  | |  | |  | |  | |  | |  |  |
| Insertion | L1650+137X |  | | | 1650 | | LP | | - | | - | |  | |  | | - | |  | |  | |  | |  | |  | |  | |  | |  | |  | |  |  |
| Missense | I1660V |  | | | 1660 | | VUS | | - | | + | | + | | + | | - | | - | | - | | - | | - | | + | | 0.1 | |  | |  | |  | |  |  |
| Missense | I1660S |  | | | 1660 | | VUS | |  | |  | |  | |  | | - | |  | |  | |  | |  | |  | |  | |  | |  | |  | |  |  |
| Missense | G1661R |  | | | 1661 | | VUS | | - | | + | | + | | + | | - | | - | | - | | - | | - | |  | | 0.05 | |  | | No effect | |  | |  |  |
| Missense | V1667I |  | | | 1667 | | VUS | | + | | + | | + | |  | | - | |  | |  | |  | |  | | + | | 1.5 | |  | |  | | 0 | | 6 |  |
| Missense | S1672Y |  | | | 1672 | | VUS | |  | |  | |  | |  | | - | |  | |  | |  | |  | |  | | 0 | |  | |  | |  | |  |  |
| Frameshift | A1680fs+106X |  | | | 1680 | | P | | + | | + | | + | | + | | - | | - | | - | | - | | - | |  | | 0 | |  | |  | |  | |  |  |
| Missense | A1680T |  | | | 1680 | | VUS | | - | | + | | + | |  | | - | | - | | - | | - | | - | |  | | 0.9 | |  | | No effect | | -5 | | -1 | No effect |
| Missense | D1690N |  | | | 1690 | | LP | | - | | + | | + | | + | | - | | - | | - | | - | | - | |  | | 0.25 | |  | | No effect | | 7 | | 0 | Slower |
| Deletion | D1690del |  | | | 1690 | | VUS | |  | |  | |  | |  | | - | |  | |  | |  | |  | |  | |  | |  | |  | |  | |  |  |
| Frameshift | D1690HfsX98 | c.5068_5070delGA | | | 1690 | | LP | |  | |  | |  | |  | | - | |  | |  | |  | |  | |  | |  | |  | |  | |  | |  |  |
| Premature truncation | Q1695X |  | | | 1695 | | VUS | | + | | + | |  | | + | | + | |  | |  | |  | |  | |  | |  | |  | |  | |  | |  |  |
| Missense | A1698T |  | | | 1698 | | VUS | |  | |  | |  | |  | | - | |  | |  | |  | |  | |  | |  | |  | |  | |  | |  |  |
| Missense | Q1706H |  | | | 1706 | | VUS | | - | | - | | - | | + | | - | | - | | - | | - | | - | |  | |  | |  | |  | |  | |  |  |
| Missense | T1709M |  | | | 1709 | | VUS | | + | | - | | + | |  | | - | | - | | - | | - | | - | |  | | 0.25 | |  | | No effect | | 1 | | 4 | Faster |
| Missense | T1709R |  | | | 1709 | | VUS | |  | |  | |  | |  | | - | |  | |  | |  | |  | |  | |  | |  | |  | |  | |  |  |
| Deletion | T1709del |  | | | 1709 | | VUS | |  | |  | |  | |  | | - | |  | |  | |  | |  | |  | |  | |  | |  | |  | |  |  |
| Frameshift | A1711Pfs | c.5131delG | | | 1711 | | LP | |  | |  | |  | |  | | - | |  | |  | |  | |  | |  | |  | |  | |  | |  | |  |  |
| Missense | G1712S |  | | | 1712 | | VUS | |  | |  | |  | |  | | - | |  | |  | |  | |  | |  | |  | |  | |  | |  | |  |  |
| Missense | D1714G |  | | | 1714 | | LP | | - | | - | | - | | + | | + | | - | | + | | + | | + | | + | | 0.2 | |  | |  | | 0 | | 0 | No effect |
| Missense | L1717P |  | | | 1717 | | VUS | | - | | - | |  | |  | | - | |  | |  | |  | |  | |  | |  | |  | |  | |  | |  |  |
| Frameshift | I1720SfsX67 | c.5157delC | | | 1720 | | LP | |  | |  | |  | |  | | - | |  | |  | |  | |  | |  | |  | |  | |  | |  | |  |  |
| Missense | N1722D |  | | | 1722 | | VUS | | + | | - | | + | | + | | + | | - | | + | | - | | - | |  | | 0.35 | |  | | Slower | | 7 | | 6 | Faster |
| Missense | P1725L |  | | | 1725 | | VUS | | - | | - | |  | | + | | - | |  | |  | |  | |  | |  | |  | |  | |  | |  | |  |  |
| Missense | C1728R |  | | | 1728 | | VUS | |  | |  | |  | |  | | - | |  | |  | |  | |  | |  | |  | |  | |  | |  | |  |  |
| Missense | C1728W |  | | | 1728 | | VUS | |  | |  | |  | |  | | - | |  | |  | |  | |  | |  | |  | |  | |  | |  | |  |  |
| Missense | P1730H |  | | | 1730 | | VUS | | + | | - | | + | | + | | - | | - | | - | | - | | - | |  | | 0.45 | |  | | Slower | | 7 | | 7 | Faster |
| Missense | G1740R |  | | | 1740 | | LP | | - | | - | | - | | - | | - | | - | | - | | - | | - | |  | | 0 | | 0.5 | | No effect | | 0 | | 0 | No effect |
| Missense | D1741Y |  | | | 1741 | | VUS | |  | |  | |  | |  | | - | |  | |  | |  | |  | |  | |  | |  | |  | |  | |  |  |
| Missense | G1743R |  | | | 1743 | | LP | | + | | + | | + | | + | | + | | + | | + | | - | | + | |  | | 0 | | 0.5 | |  | | 0 | | 0 | Slower |
| Missense | G1743E |  | | | 1743 | | LP | | + | | - | | + | | + | | - | | - | | - | | - | | - | | + | | 0.05 | |  | |  | |  | |  |  |
| Missense | G1748D |  | | | 1748 | | LP | | - | | + | | + | | + | | - | | - | | - | | - | | - | |  | | 0.1 | | 0.2 | | No effect | | 13 | | 14 | Faster |
| Frameshift | c.5280delG |  | | | 1761 | | LP | |  | |  | |  | |  | | - | |  | |  | |  | |  | |  | |  | |  | |  | |  | |  |  |
| Missense | V1764D |  | | | 1764 | | VUS | | + | | - | |  | |  | | - | |  | |  | |  | |  | |  | |  | |  | |  | |  | |  |  |
| Missense | V1764F |  | | | 1764 | | VUS | | - | | - | | - | | + | | - | | - | | - | | - | | - | |  | |  | |  | |  | |  | |  |  |
| Frameshift | V1764SfsX23 | c.5290delG | | | 1764 | | LP | |  | |  | |  | |  | | - | |  | |  | |  | |  | |  | |  | |  | |  | |  | |  |  |
| Missense | M1766T |  | | | 1766 | | VUS | |  | |  | |  | |  | |  | |  | |  | |  | |  | |  | |  | |  | |  | |  | |  |  |
| Frameshift | L1772WfsX15 | c.5314delC | | | 1772 | | LP | |  | |  | |  | |  | | - | |  | |  | |  | |  | |  | |  | |  | |  | |  | |  |  |
| Insertion | N1774+12X |  | | | 1774 | | LP | | + | | - | | + | | - | | + | | - | | - | | - | | + | |  | | 0 | | 0.5 | |  | |  | |  |  |
| Frameshift | c.5321_5324dupACTT | 1774 | VUS |  | |  | |  | |  | | + | |  | |  | |  | |  | |  | |  | |  | |  | |  | |  | |  | |  |  |  |
| Missense | T1779M |  | | | 1779 | | VUS | |  | |  | |  | |  | | - | |  | |  | |  | |  | |  | |  | |  | |  | |  | |  |  |
| Missense | E1780G |  | | | 1780 | | VUS | |  | |  | |  | |  | | - | |  | |  | |  | |  | |  | |  | |  | |  | |  | |  |  |
| Missense | E1784K |  | | | 1784 | | LP | | + | | + | | + | | + | | + | | + | | - | | - | | - | | + | | 0.4 | | 0.7 | | Faster | | 9 | | -18 | Faster |
| Frameshift | L1786EfsX2 | c.5356_5357delCT | | | 1786 | | P | | + | | - | | + | | + | | + | | + | | - | | - | | - | |  | | 0 | |  | |  | |  | |  |  |
| Missense | L1786Q |  | | | 1786 | | LP | | + | | + | | + | | - | | - | | - | | - | | - | | - | | + | | 0.3 | |  | |  | | 15 | | -21 |  |
| Missense | S1787N |  | | | 1787 | | VUS | |  | |  | |  | |  | |  | |  | |  | |  | |  | |  | |  | |  | |  | |  | |  |  |
| Missense | D1790G |  | | | 1790 | | VUS | |  | |  | |  | |  | |  | |  | |  | |  | |  | |  | |  | |  | |  | |  | |  |  |
| Insertion | 1795insD |  | | | 1795 | | LP | | + | | + | | + | | + | | + | | + | | + | | - | | + | | + | | 0.2 | |  | | No effect | | 8 | | -7 | Slower |
| Missense | Y1795H |  | | | 1795 | | LP | | - | | - | | - | | + | | - | | - | | - | | - | | - | |  | | 0.5 | |  | | Faster | | 0 | | -11 | No effect |
| Frameshift | F1808IfsX2 | c.5420dupA | | | 1808 | | LP | |  | |  | |  | |  | | - | |  | |  | |  | |  | |  | |  | |  | |  | |  | |  |  |
| Premature truncation | S1812X |  | | | 1812 | | P | | + | | + | |  | | - | | + | |  | |  | |  | |  | |  | | 0.05 | |  | |  | |  | |  |  |
| Frameshift | D1816VfsX7 |  | | | 1816 | | P | | + | | - | | + | | - | | + | | + | | - | | - | | + | |  | | 0.1 | |  | | No effect | | 18 | | 20 | Faster |
| Frameshift | E1823HfsX10 | c.5464_5467delTCTG | | | 1823 | | LP | |  | |  | |  | |  | | - | |  | |  | |  | |  | |  | |  | |  | |  | |  | |  |  |
| Missense | Q1832E |  | | | 1832 | | VUS | |  | |  | |  | |  | | - | |  | |  | |  | |  | |  | | -0.55 | |  | | No effect | | 0 | | 0 |  |
| Missense | R1847H |  | | | 1847 | | VUS | |  | |  | |  | |  | | - | |  | |  | |  | |  | |  | |  | |  | |  | |  | |  |  |
| Missense | C1850S |  | | | 1850 | | LP | | - | | + | | + | | + | | - | | - | | - | | - | | - | |  | | 0.4 | | 0.8 | | Faster | | 0 | | -12 | No effect |
| Frameshift | R1860KfsX13 | c.5577_5578dupAA | | | 1860 | | LP | |  | |  | |  | |  | | - | |  | |  | |  | |  | |  | |  | |  | |  | |  | |  |  |
| Missense | V1861I |  | | | 1861 | | VUS | |  | |  | |  | |  | | - | |  | |  | |  | |  | |  | |  | |  | |  | |  | |  |  |
| Missense | K1872N |  | | | 1872 | | VUS | |  | |  | |  | |  | | - | |  | |  | |  | |  | |  | |  | |  | |  | |  | |  |  |
| Insertion | 1876insM |  | | | 1876 | | VUS | | + | | - | | + | | + | | - | | - | | - | | - | | - | |  | | 1 | |  | | Slower | | 8 | | -8 | Slower |
| Missense | M1880V |  | | | 1880 | | VUS | | - | | - | | - | | - | | + | | + | | - | | - | | - | |  | | 1 | |  | |  | | 8 | | -6 | No effect |
| Frameshift | T1893PfsX29 | c.5676delC | | | 1893 | | LP | |  | |  | |  | |  | |  | |  | |  | |  | |  | |  | |  | |  | |  | |  | |  |  |
| Missense | R1898C |  | | | 1898 | | VUS | | - | | - | | - | | - | | - | | - | | - | | - | | - | |  | | 0.3 | |  | | No effect | | -8 | | -5 | Slower |
| Missense | S1904L |  | | | 1904 | | VUS | |  | |  | |  | |  | | - | |  | |  | |  | |  | |  | |  | |  | |  | |  | |  |  |
| Missense | R1913H |  | | | 1913 | | VUS | |  | |  | |  | |  | | - | |  | |  | |  | |  | |  | |  | |  | |  | |  | |  |  |
| Missense | R1913C |  | | | 1913 | | VUS | | - | |  | |  | |  | |  | |  | |  | |  | |  | |  | |  | |  | |  | |  | |  |  |
| Missense | R1919H |  | | | 1919 | | VUS | | - | |  | |  | |  | |  | |  | |  | |  | |  | |  | |  | |  | |  | |  | |  |  |
| Premature truncation | L1921X |  | | | 1921 | | LP | |  | |  | |  | |  | | - | |  | |  | |  | |  | |  | |  | |  | |  | |  | |  |  |
| Missense | H1923D |  | | | 1923 | | VUS | | - | |  | |  | |  | |  | |  | |  | |  | |  | |  | |  | |  | |  | |  | |  |  |
| Missense | A1924T |  | | | 1924 | | VUS | | + | | - | | + | | + | | + | | + | | - | | - | | - | |  | | 1 | |  | | Faster | |  | | 0 |  |
| Missense | R1929C |  | | | 1929 | | VUS | |  | |  | |  | | + | | - | |  | |  | |  | |  | |  | |  | |  | |  | |  | |  |  |
| Missense | G1935S |  | | | 1935 | | LB | |  | |  | |  | |  | | - | |  | |  | |  | |  | |  | |  | |  | |  | |  | |  |  |
| Missense | S1937F |  | | | 1937 | | VUS | | + | | - | |  | |  | | - | |  | |  | |  | |  | |  | |  | |  | |  | |  | |  |  |
| Missense | E1938K |  | | | 1938 | | VUS | |  | |  | |  | |  | | - | |  | |  | |  | |  | |  | | 0 | | 0.3 | |  | |  | | 0 |  |
| Missense | A1949P |  | | | 1949 | | VUS | | - | | - | | - | | + | | - | | - | | - | | - | | - | |  | |  | |  | |  | |  | |  |  |
| Missense | V1951M |  | | | 1951 | | LB | | - | | + | | + | | - | | - | | - | | - | | - | | - | |  | |  | |  | |  | |  | |  |  |
| Missense | V1951L |  | | | 1951 | | VUS | |  | |  | |  | |  | |  | |  | |  | |  | |  | |  | |  | |  | |  | |  | |  |  |
| Missense | S1964F |  | | | 1964 | | VUS | |  | |  | |  | |  | | - | |  | |  | |  | |  | |  | |  | |  | |  | |  | |  |  |
| Missense | I1968S |  | | | 1968 | | VUS | | + | | - | | + | | + | | - | | - | | - | | - | | - | |  | | 0.65 | |  | | Faster | | 0 | | 0 | Faster |
| Missense | L1988R |  | | | 1988 | | VUS | |  | |  | |  | |  | |  | |  | |  | |  | |  | |  | |  | |  | |  | |  | |  |  |
| Duplication | F2004dup |  | | | 2004 | | VUS | |  | |  | |  | |  | | - | |  | |  | |  | |  | |  | |  | |  | |  | |  | |  |  |
| Missense | F2004V |  | | | 2004 | | VUS | |  | |  | |  | |  | | - | |  | |  | |  | |  | | + | |  | |  | |  | |  | |  |  |
| Missense | F2004L |  | | | 2004 | | VUS | | - | | - | | - | | - | | + | | - | | + | | - | | + | |  | | 0.55 | |  | |  | | 0 | | -5 | Slower |
| Missense | P2006A |  | | | 2006 | | VUS | |  | |  | |  | |  | |  | |  | |  | |  | |  | |  | |  | |  | |  | |  | |  |  |
| Missense | R2012H |  | | | 2012 | | VUS | | - | | + | | + | | + | | - | | - | | - | | - | | - | |  | | 0.75 | |  | | Faster | | 0 | | -2 | No effect |
| Missense | V2016M |  | | | 2016 | | VUS | | - | | - | | - | | + | | - | | - | | - | | - | | - | |  | |  | | 0.65 | |  | | 0 | | 0 |  |
